# Design strategies, manufacturing, and applications of radiative cooling technologies

**DOI:** 10.1515/nanoph-2025-0159

**Published:** 2025-07-02

**Authors:** Joonho Kang, Changkyun Lee, Haejun Chung, Peter Bermel

**Affiliations:** Department of Artificial Intelligence Semiconductor Engineering, Hanyang University, Seoul, 04763, South Korea; Education and Research Group Fostering Convergence IT Engineers, Hanyang University, Seoul, 04763, South Korea; Department of Electronic Engineering, Department of Artificial Intelligence, and Department of Artificial Intelligence Semiconductor Engineering, Hanyang University, Seoul, 04763, South Korea; Birck Nanotechnology Center, and Elmore Family School of Electrical and Computer Engineering, 311308 Purdue University, West Lafayette, IN, 47907, USA

**Keywords:** radiative cooling, nanophotonics, design strategies, energy efficiency, sustainable cooling technologies

## Abstract

Radiative cooling is a passive cooling strategy that leverages thermal radiation to dissipate heat into a cooler environment, offering an energy-efficient and environmentally friendly alternative to conventional cooling technologies. Recent advancements in material science and nanophotonics have led to the development of engineered radiative cooling materials with tailored optical and thermal properties. Photonic structures, multilayer films, metamaterials, and polymer-based composites have demonstrated enhanced cooling performance by maximizing solar reflectance and infrared emissivity. These innovations have facilitated scalable, lightweight, and durable cooling solutions suitable for diverse applications, including building envelopes, electronic devices, and urban infrastructure. Nonetheless, several challenges must be solved to achieve widespread commercialization. These include further research into robust and long-lasting materials to address material degradation, innovations in fabrication techniques to reduce cost, design approaches to make more effective use of these materials and processes, and adaptability to hot and humid climates. Ongoing research continues to refine material and structural design, improve manufacturing methods, and expand the range of practical applications. By overcoming these challenges, radiative cooling has the potential to significantly reduce energy consumption and enhance climate resilience, positioning itself as a crucial component of future sustainable cooling technologies.

## 1 Introduction

Radiative cooling is a passive method that operates by emitting thermal radiation into a cooler environment. Unlike conventional cooling technologies that rely on electricity or refrigerants, radiative cooling presents an energy-efficient, environmentally friendly, and long-lasting solution for temperature regulation in buildings [1], [2], [3], electronic devices [4], [5], [6], [7], [8], and other applications [9], [10], [11], [12], [13], [14]. It generally requires no input power to operate and can operate under the right environment, without any moving parts.

There are two major types: above-ambient [15], [16] and subambient cooling [17], [18], [19]. Above-ambient cooling [20], [21], [22] operates across a broad range of wavelengths, but typically the most relevant wavelengths are centered around the thermal emission peak, which generally is around 10 μm at room temperature. Broadband emitters are particularly effective for above-ambient cooling because their emission spectra significantly overlap with the blackbody radiation spectrum at ambient temperature. At around 300 K, blackbody radiation is concentrated between 8 and 26 µm [23]. Therefore, broadband emitters that exhibit high emissivity across this infrared range can efficiently radiate thermal energy to the atmosphere and outer space, enabling effective heat emission even when the emitter’s temperature is above ambient.

On the other hand, subambient cooling emits through the atmospheric transparency window (8–13 µm) [24], [25], [26], [27], allowing heat to escape into space. To achieve subambient temperatures under direct sunlight, materials must be engineered to exhibit high reflectivity in the solar spectrum (0.3–2.5 µm) while simultaneously demonstrating strong infrared emissivity within the atmospheric transparency window. Selective emitters, introduced by Grenier [28], are typically employed for this purpose, as they are designed to suppress absorption of incoming solar radiation while enhancing emission within the atmospheric transparency window region. This spectral selectivity is beneficial for enabling daytime radiative cooling below ambient temperature. Such a situation ensures minimal solar heat gain and maximized thermal emission. The interplay between these two optical properties dictates the effectiveness of radiative cooling systems, influencing their viability across various environmental conditions. Generally, this approach is most viable in hot and dry climates because the temperature difference and transparency in the long wavelength infrared range are both maximized.

Recent advancements in material science and nanophotonics have enabled the development of specialized radiative cooling materials with tailored optical [29], [30], [31], thermal [32], [33], [34], and mechanical [35], [36], [37] properties. Photonic structures, including multilayer films [38], [39], metamaterials [40], [41], and periodic nanostructures [23], allow precise control over spectral properties to achieve optimal reflectance and emittance. These engineered materials have demonstrated significant cooling power by minimizing solar absorption while enhancing thermal radiation. Polymers structure [42], [43], [44] have also emerged as promising solutions, incorporating nanoparticle inclusions [45], [46] or hierarchical microstructures [47] to reflect solar radiation efficiently while maintaining high infrared emissivity. They facilitate efficient all-day passive radiative cooling, maintaining subambient temperatures even under direct sunlight. Their lightweight and scalable manufacturing make them suitable for large-scale cooling applications. Additionally, the integration of radiative cooling principles into building materials [2], [48], [49] has led to innovations such as cooling wood, which exhibits modified cellulose structures to enhance infrared emission while reflecting solar radiation. Studies have demonstrated energy savings ranging from 20 % to 60 % in hot and dry climates [50], [51], [52], [53], [54], underscoring the potential of radiative cooling materials in sustainable architecture and urban planning.

The continuous evolution of radiative cooling technologies holds significant promise for mitigating energy consumption and addressing global climate challenges. By refining material designs and expanding their practical applications, researchers aim to enhance the efficiency [21], [55], [56], durability [49], [57], [58], [59], and scalability [59], [60] of radiative cooling solutions. This review provides a comprehensive analysis of recent advancements in radiative cooling materials, focusing on their design principles, performance metrics, and emerging applications in real-world settings.

## 2 Historical overview of radiative cooling

The concept of radiative cooling may have originally been used in ancient times to form ice and has been recognized in the scientific literature since the early 20th century.

Between the 1920s and 1940s, researchers began to investigate the principles of thermal radiation and its role in cooling surfaces exposed to the sky. Early scientific studies focused on understanding the radiative heat transfer mechanisms and their potential applications in passive cooling strategies [61], [62], [63], [64]. Observations of nocturnal cooling effects in dry and high-altitude environments [65], [66] further spurred interest in utilizing radiative cooling for temperature regulation.

During the latter half of the 20th century, researchers explored practical applications of radiative cooling. By the 1970s, scientists started experimenting with bulk materials that could enhance infrared emission while minimizing solar absorption [67], [68], [69]. The energy crisis of the 1970s also accelerated interest in passive cooling solutions, prompting the development of early radiative cooling surfaces for buildings and industrial applications [70], [71], [72]. Subsequent studies in the 1980s and 1990s laid the foundation for modern advancements by characterizing the spectral properties of various materials and their effectiveness in radiative cooling [73], [74], [75], [76], [77].

Modern advancements in radiative cooling since the beginning of the 21st century have focused on developing spectrally selective coatings and advanced material engineering. The last two decades have seen significant progress in nanophotonic designs [78], [79], [80], [81], multilayer coatings [38], [82], [83], [84], and metamaterials [80], [85], [86], [87] that optimize the balance between solar reflectivity and infrared emissivity. These developments have enabled radiative cooling to be effectively integrated into a variety of practical applications. One of the most impactful applications is building thermal management, where radiative cooling materials have been applied to roofs and walls to reduce indoor temperatures, decreasing reliance on air conditioning and contributing to energy savings [2], [48], [49], [88]. Semiconductor device cooling has also benefited from radiative cooling, as it aids in maintaining optimal operating temperatures for electronics, including photovoltaics and other temperature-sensitive systems, thereby enhancing performance and prolonging device lifespan [21], [55], [89], [90], [91]. Additionally, wearable radiative cooling materials have been explored to provide personal thermal comfort without external energy input [58], [59], [92], [93], [94]. Radiative cooling has also found use in food and water applications, particularly when combined with water condensation techniques to improve the efficiency of water and food collection systems and regulate temperatures in agricultural environments [95], [96], [97].

## 3 Design strategies

**Figure 1:**
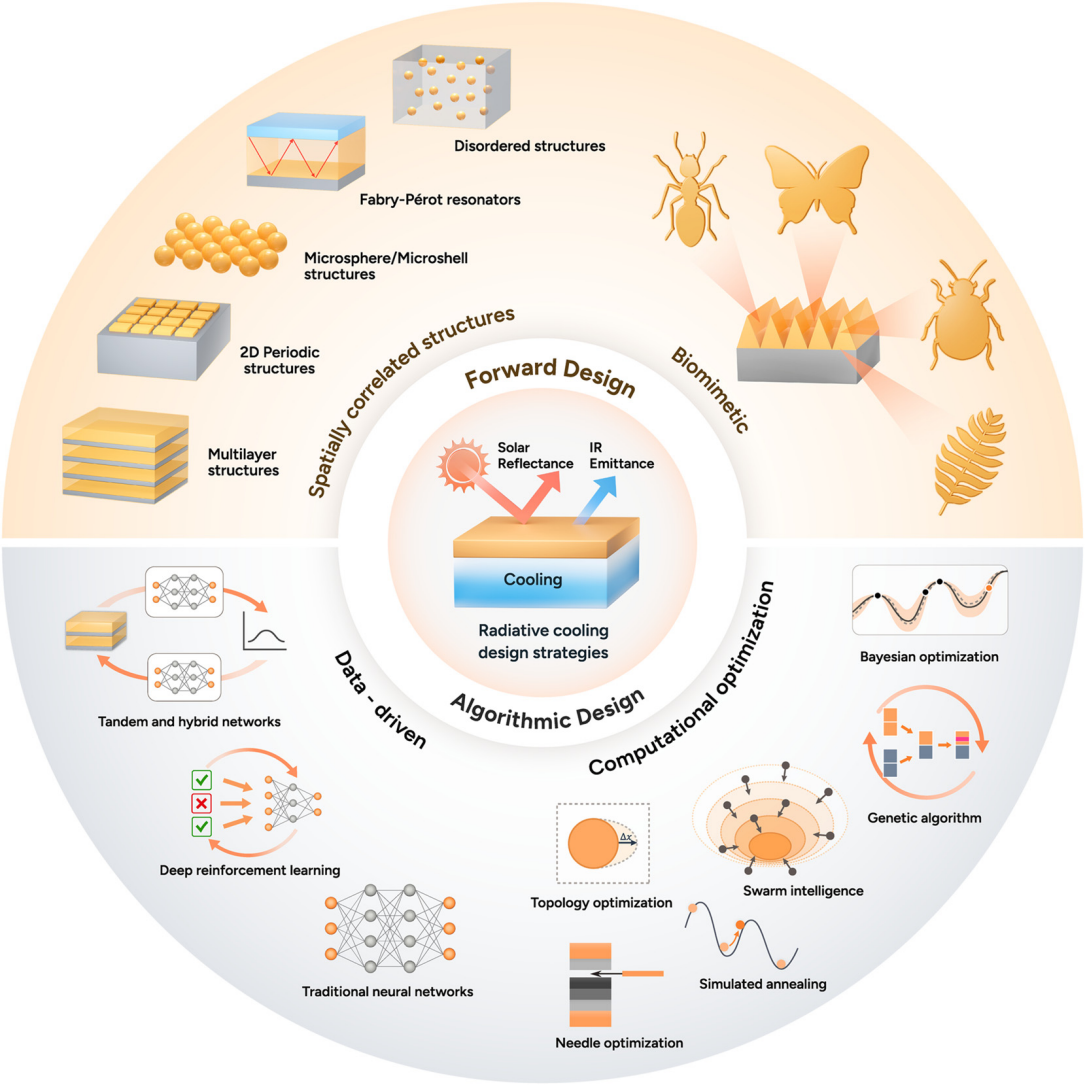
Overview of the key design strategies in radiative cooling.

The design strategies of radiative cooling have been evolved by advancements in material science, computational techniques, and bio-inspired engineering. Early studies focused on utilizing natural materials with high infrared emissivity [68], [72], [98], but modern approaches have leveraged structured photonics [20], [78], [80] and computational design [99], [100], [101] to enhance cooling performance. Initially, bulk materials with strong thermal emissivity [19], [102] were explored, but the development of multilayer thin films [103], [104], [105], photonic crystals [16], [106], and metamaterials [87], [107], [108] allowed precise spectral control. These designs exploit interference effects and resonances to maximize cooling while minimizing solar absorption. Nature has long provided efficient cooling mechanisms. Inspired by desert beetles [109], butterfly wings [110], and plant structures [111], recent studies have designed surfaces that selectively reflect solar radiation while enhancing infrared emission. Such bio-inspired approaches gained traction as fabrication techniques improved in recent decades. As material complexity increased, computational methods became essential for refining designs. Genetic algorithms [112], [113], swarm intelligence [114], [115], [116], and topology optimizations [117] now enable highly efficient cooling structures that are unlikely to be discovered through trial-and-error approaches. In recent years, machine learning and artificial intelligence algorithms have been used to discover material and inverse design. Artificial intelligence-based models [118], [119], [120] predict optimal structures or thermal properties, accelerating the development of high-performance cooling materials beyond traditional theoretical models. These evolving design methodologies continue to push the boundaries of radiative cooling, enabling more efficient and scalable applications. Figure 1 provides an overview of these design strategies, illustrating the key approaches discussed in this review. The following sections provide a detailed discussion of each approach.

### 3.1 Spatially correlated structure-based approach

One powerful method in achieving radiative cooling is the precise control of a material’s structure. Here, the main goal is to engineer structures based on established physical theories and mechanisms so that solar spectra are strongly reflected while the longer-wavelength thermal emissions, especially around 8–13 µm, escape freely into the colder sky. Spatially correlated design methods achieve this by arranging, or sometimes even randomly dispersing, structures such as layered films, metasurfaces, or porous networks to generate desired optical effects. In the following, we discuss how each structure contributes to radiative cooling through its theoretical principles.

**Figure 2:**
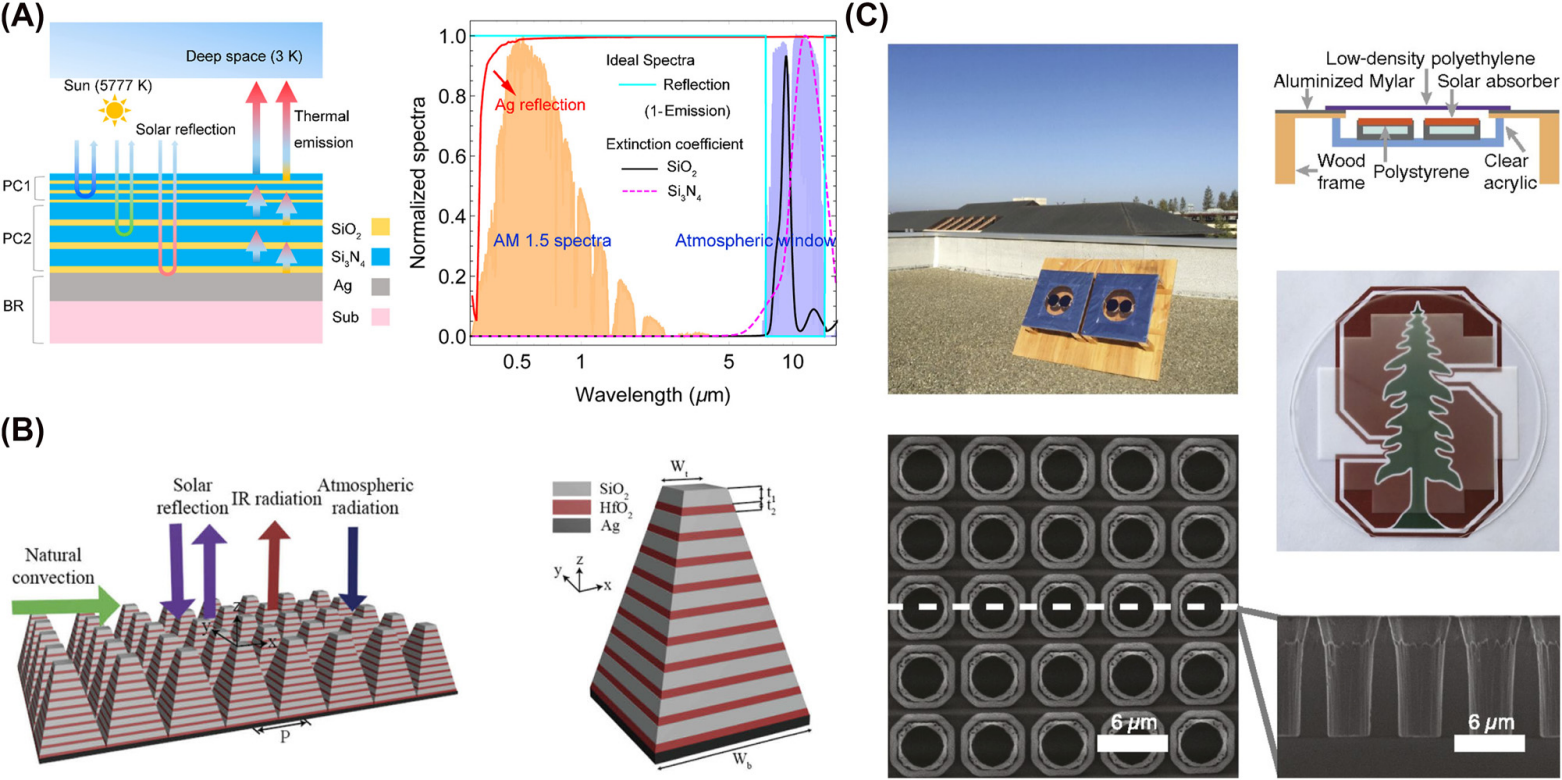
Examples of multilayer and 2D periodic structures for radiative cooling. (A) A tandem 1D photonic crystal is designed by utilizing quarter-wave stacks, efficiently reflecting solar radiation while emitting thermal spectrum. Reprinted with permission from [121]. Copyright 2019 American Chemical Society. (B) A periodic metasurface with pyramidal multilayer structure with alternating HfO_2_ and SiO_2_ layers is designed, enhancing IR absorptivity through multiple resonances and moth-eye-inspired antireflection effect. Adapted with permission from [23] © Optica Publishing Group. (C) A 2D photonic crystal introduces a gradual refractive index transition, effectively matching impedance between silica and air over thermal wavelengths and yielding near-unity emissivity when placed atop a silicon absorber. Reproduced with permission from [122].

#### 3.1.1 Multilayer thin-film structures

Multilayer thin-films are composed of alternating layers of two materials each with different refractive indices, vertically stacked [123]. When the thicknesses of these layers follow a periodic pattern, the structure is classified as a one-dimensional (1D) photonic crystal [123], [124], the simplest form of highly ordered structures. In this context, the terms “multilayer thin-film” and “1D photonic crystal” can be used interchangeably.

A fundamental characteristic of wave propagation in structured media is the dispersion relation. In an isotropic, homogeneous, and dispersionless medium, the relationship between frequency and momentum of waves is linear, resulting in straight curves in the photonic band structure. However, when a periodic structure such as a 1D photonic crystal is introduced, it functions as a Bragg reflector, enabling high reflectivity within specific narrow or multiple spectral bands [125]. As a result, such periodic structures selectively permit the transmission of certain frequencies of light while preventing the propagation of others. As such, the primary design strategy of incorporating 1D photonic crystals is to induce a photonic bandgap effect to manipulate reflection and emissivity to realize radiative cooling.

To implement such a strategy, quarter-wavelength stacks [126] are commonly employed. The design principle of this structure is that the optical thickness of each layer *t*_*i*_ should be a quarter of the target center wavelength *λ*_*c*_. The thickness of each layer is given by:

(1) $$t_{i}=\frac{\lambda_{c}}{4n_{i}}$$

where *n*_*i*_ is the real part of the complex refractive index of the material. In the study by [121], this strategy was applied to engineer a tandem structure of silicon dioxide (SiO_2_)/silicon nitride (Si_3_N_4_) 1D photonic crystals to achieve broadband reflection in the solar spectrum while exhibiting strong thermal emission in the MIR atmospheric window, as shown in Figure 2A. This strategy is also used in the development of a resonant-cavity enhanced narrowband thermal emitter [127], a broadband angle selective low-pass filter [128], and a laser-processed surface with tailored spectral and directional emissivity for radiative cooling and thermal emission control [129].

While many multilayer structures for radiative cooling are fabricated using alternating dielectric films, alternatives using polymeric multilayers have also emerged. Gentle and Smith [130] demonstrated subambient daytime cooling using a commercially available coextruded polymer multilayer film, consisting of over 300 graded bilayers of PET and ECDEL with a silver backing. The stack functions as a 1D dielectric mirror with high solar reflectance and strong thermal emissivity in the 8–13 µm range, enabling continuous subambient cooling even under 1,060 W m^−2^ solar irradiance without a convention barrier. Unlike traditional inorganic photonic crystals, this approach utilizes scalable polymer processing, demonstrating potential compatibility with building-integrated cooling applications.

The key advantage of these multilayer thin-film structures is their high spectral selectivity [131], [132], [133], as well as high-throughput manufacturing owing to lithography-free fabrication [134]. However, a possible limitation of these multilayer thin-film systems is that their performance is usually sensitive to the incidence angle [135]. To address this, multiple photonic crystal layers with different periodicities can be stacked in tandem, as addressed in [121], or an increased number of layers can be used to widen the bandgap. However, these approaches can increase the overall bulkiness of the structure, leading to fabrication challenges and potential optical losses due to imperfections in the multilayer stack.

#### 3.1.2 2D periodic structures

Two-dimensional (2D) periodic structures, such as photonic crystals and metasurfaces, have emerged as powerful tools for tailoring thermal radiation. By leveraging periodicity at the subwavelength scale in two dimensions, these structures provide precise control over spectral emissivity with higher degrees of freedom.

Metasurfaces are engineered surfaces consisting of subwavelength periodic structures that can control the phase, amplitude, or polarization of light [136]. With this, they achieve spectral responses that are otherwise difficult to realize with homogeneous materials or conventional materials found in nature [137], [138]. Along with 2D photonic crystals, metasurfaces, especially 2D periodic metasurfaces, have emerged as a prominent strategy for tailoring emissivity and suppressing unwanted solar absorption by carefully designing their nanostructured unit cells, or meta-atoms. For instance, a metamaterial-based thermal emitter [85] can be engineered using an anisotropic conical-shaped metamaterial structure, where each meta-atom consists of an Al–Ge multilayer arranged in a conical geometry, ensuring polarization insensitivity and broadband thermal emission. An ultra-broadband all-dielectric metamaterial thermal emitter [23] can also be developed using a pyramidal multilayer structure composed of alternating HfO_2_ and SiO_2_ layers to enhance IR absorptivity through multiple resonances and moth-eye-inspired antireflection effects, as shown in Figure 2B. Similarly, a metal–insulator–metal nano-infrared emitter [139] can be designed with a diamond-shaped metasurface grating, which exploits surface plasmon polaritons, FP resonances, and impedance matching to achieve multiband selective IR absorption.

2D photonic crystals, unlike 1D photonic crystals, introduce periodicity in two dimensions [124]. While conventional photonic crystals use Bragg scattering to control emissivity, radiative cooling applications exploit other physical mechanisms within photonic crystal structures to tailor thermal emission. For example, a transparent photonic crystal thermal blackbody [122] is created by etching air holes with nonvertical sidewalls into a silica wafer, as shown in Figure 2C. This design enables a gradual refractive index transition, ensuring effective impedance matching between silica and air across thermal wavelengths, resulting in near-unity emissivity when placed atop a silicon absorber. Similarly, a metallodielectric two-dimensional photonic crystal [140] is designed with tantalum cavities that are filled and capped with hafnium dioxide (HfO_2_). This configuration enhances its performance as a selective emitter by maximizing in-band emissivity while suppressing out-of-band radiation. The filled cavities and precisely controlled capping layer provide spectral selectivity, ensuring thermal radiation.

2D periodic metasurfaces and photonic crystals can control the spectral response at subwavelength scales. Also, being thin and planar, they are relatively easy to integrate with existing technologies as coatings. However, high-precision nanofabrication, such as lithography [141], is required, as the performance of these structures is highly dependent on the exact dimensions and periodicity of the nanostructures. Despite these limitations, these diverse photonic architectures significantly expand the design space for high-performance, passive cooling technologies.

**Figure 3:**
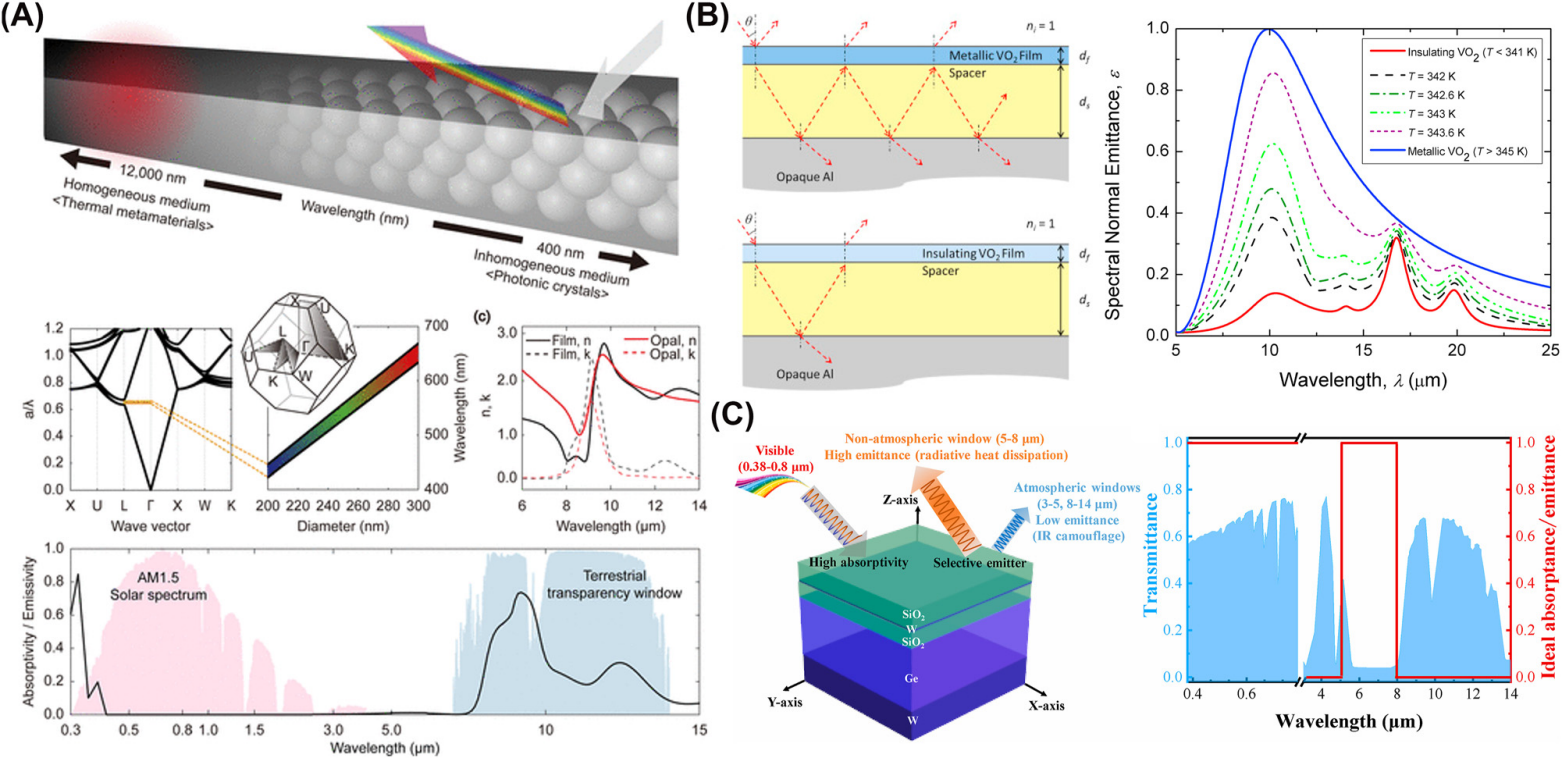
Examples of microsphere structures and Fabry–Pérot resonators. (A) A self-assembled opaline structure of 300 nm silica nanospheres is arranged in a face-centered-cubic lattice. This configuration exhibits Bragg scattering in the visible spectrum, producing structural coloration, and simultaneously serves as an effective medium for strong thermal emission conducive to radiative cooling. Adapted with permission from [142]. Copyright 2020 American Chemical Society. (B) An asymmetric FP resonator is formed by a metallic VO_2_ and an opaque Al substrate, separated by a dielectric spacer. Above 345 K, metallic VO_2_ forms a resonant cavity enabling strong MIR emission around 10 µm, whereas below 341 K, insulating VO_2_ minimizes thermal radiation losses [143]. Reproduced with permission, Copyright 2017 Elsevier. (C) A thick film W/Ge/SiO_2_/ultrathin film W/SiO_2_ multilayer system [144] is engineered as a dual-dielectric FP resonator that balances IR stealth and radiative heat dissipation. In this design, the Ge and SiO_2_ dielectric layers provide spectral selectivity, the ultrathin W layer enables resonant tunneling for enhanced absorption and emission, and the thick W bottom layer ensures high reflectivity within the infrared atmospheric windows. Reproduced with permission, Copyright 2025 Elsevier.

#### 3.1.3 Microsphere and microshell structures

Not all photonic designs require long-range periodicity to achieve strong solar reflection and robust MIR emission. Arrays of microspheres and microshells or even carefully tuned optical cavities can also provide the necessary spectral control. A common strategy is embedding spherical or shell-like inclusions (e.g., SiO_2_, TiO_2_, or doped metal oxides) in a polymer or glassy matrix [145], [146], [147].

In particular, microsphere and shell-based photonic crystals [148] enhance MIR emissivity by efficiently outcoupling surface phonon–polariton into free space, for strong thermal emission within the atmospheric transparency window. They also reduce reflection losses compared to bulk silica by suppressing the Restrahlen band. The self-assembled opaline structure [142] can be formed with 300 nm silica nanospheres arranged in a face-centered-cubic lattice, thus, exhibiting Bragg scattering in the visible spectrum, leading to structural coloration, as shown in Figure 3A. The opals also act as an effective medium, simultaneously enabling strong thermal radiation for cooling. A plasmonic nanocrystal-base film [149] can also be designed, which utilizes localized surface plasmon resonance in doped metal oxide nanocrystals for infrared emission. By adjusting the nanocrystal size, doping concentration, and film thickness, the emissivity spectrum can be selectively engineered to enhance cooling efficiency while minimizing solar absorption.

While these architectures offer tunable optical properties and scalable fabrication, they also come with limitations. Their performance is highly dependent on particle size distribution and spatial arrangement [148], [149]. Thus, it is important to carefully control these factors to optimize spectral selectivity while mitigating performance trade-offs.

#### 3.1.4 Fabry–Pérot (FP) resonators

Thin-film resonant cavities, such as Fabry–Pérot (FP) resonators, can boost emission at specific IR wavelengths through constructive interference. A conventional FP resonator is made of two parallel reflectors separated at a finite distance [135], [150], where multiple internal reflections lead to constructive interference at resonant wavelengths, forming discrete transmission peaks, while destructive interference occurs at nonresonant wavelengths. However, this configuration primarily supports high-quality factor transmission modes and is commonly employed in optical filters or interferometry [151], rather than thermal emission control.

Thus, the commonly used FP resonator in radiative cooling is the asymmetric type, where one of the reflectors is replaced by a semi-infinite metallic layer [135]. This layer, with a thickness exceeding the optical penetration depth, ensures negligible transmission, making it effectively opaque. As a result, instead of supporting transmission-based resonance, the structure operates by selectively enhancing emissivity at specific wavelengths.

FP resonance is achieved when the total phase shift within the cavity is an integer multiple of 2*π*, resulting in the formation of standing waves. The work of Taylor et al. [143] mathematically describes this as:

(2) $$2\Psi =2\beta +\psi_{b}+\psi_{s}$$

where 2Ψ is the total phase shift accumulated over a full round trip within the cavity, and *ψ*_*b*_ and *ψ*_*s*_ correspond to phase angles of the reflection coefficients at the top and bottom interfaces, respectively. The phase shift traveling inside the cavity *β* is calculated with *β* = 2*πn*_*s*_ *d*_*s*_ cos*θ*_*c*_/*λ*, where *n*_*s*_ is the refractive index of the spacer material, *d*_*s*_ is the thickness of the spacer layer, *θ*_*c*_ is the internal angle of refraction, and *λ* is the wavelength of the incident wave. With this formulation, Taylor et al. verify FP resonance of their designed cavity structure, which consists of a metallic vanadium dioxide (VO_2_) film and opaque aluminum (Al) substrate separated by a spacer with a relative permittivity of 3.4, as shown in Figure 3B. A similar verification is conducted by [152] in designing a triple-layer FP absorber consisting of two silver layers separated between a dielectric spacer with a relative permittivity of 3.42.

There are many other material choices that can realize FP resonances with this structure. For example, a silicon carbide layer sandwiched between a nanoscopic platinum working electrode and a barium fluoride substrate works as a conventional FP resonator, forming a key component of a six-layer adaptive IR radiation modulator [153]. The FP resonator transmits IR light to the underlying gel-electrolyte layer, where it interacts with the reversible metal electrodeposition process, enabling dynamic control over emissivity and reflectivity. Also, a VO_2_/zinc selenide (ZnSe)/indium tin oxide (ITO)/glass multilayer system [154] functions as a thermochromic FP cavity, dynamically modulating IR emissivity by exploiting the phase transition of VO_2_ to regulate radiative cooling. A thick film tungsten (W)/germanium (Ge)/SiO_2_/ultrathin film W/SiO_2_ multilayer system [144], depicted in Figure 3C, is designed as a dual-dielectric FP resonator that balances IR stealth and radiative heat dissipation, where the germanium and SiO_2_ dielectric layers enable spectral selectivity, the ultrathin W layer facilitates resonant tunneling for enhanced absorption and emission, and the thick W bottom layer ensures high reflectivity in the infrared atmospheric windows.

These partially ordered or resonant configurations generally allow strong manipulation of specific wavelength bands with fewer overall layers or simpler geometries. These designs are less sensitive to the angle of incidence than fully periodic photonic crystals, although performance still varies with angle.

**Figure 4:**
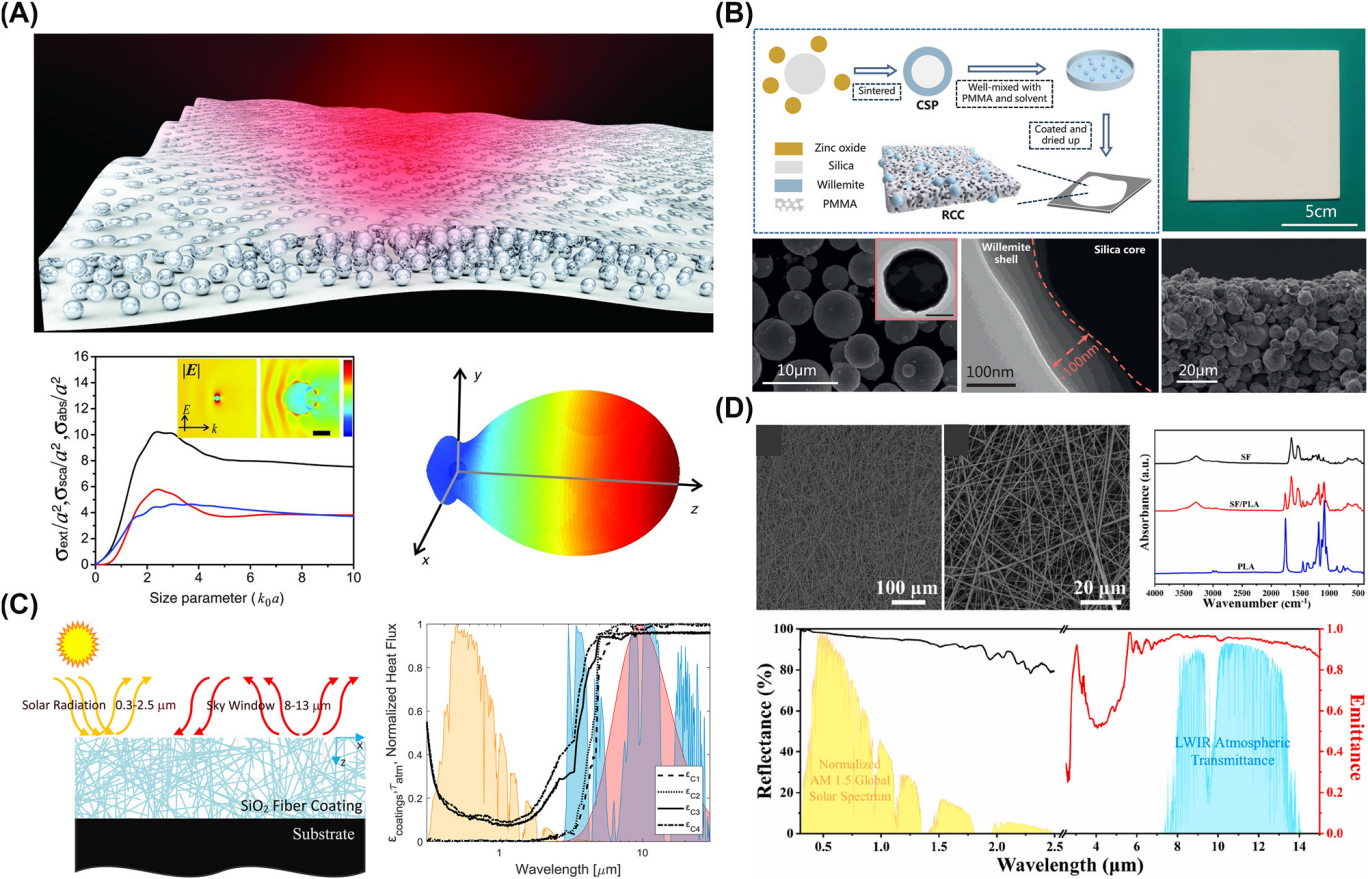
Examples of disordered structures for radiative cooling. (A) Randomly distributed silica microspheres in a polymer matrix preferentially scatter thermal infrared radiation while remaining largely transparent to visible light. From [81]. Reprinted with permission from AAAS. (B) A core–shell particle configuration boosts solar reflectance while maintaining strong MIR emission for daytime radiative cooling [155]. By engineering the shell and leveraging intrinsic phonon modes, the design achieves subambient temperatures under full sun conditions. Reproduced with permission, Copyright 2021 Elsevier. (C) A silica fiber network robustly backscatters sunlight, reducing absorbed heat and maintaining a cooler surface [156]. Simultaneously, its broadband infrared emission cuts radiative losses, enabling cooling even under direct sunlight. Reproduced with permission, Copyright 2020 Elsevier. (D) A biodegradable fiber membrane integrates high solar reflectance with strong MIR emission for eco-friendly radiative cooling [157]. Fabricated from biomass-derived polymers, it delivers tangible subambient cooling while degrading quickly in outdoor soil. Reproduced with permission, Copyright 2023 Elsevier.

#### 3.1.5 Disordered structures

In radiative cooling, structural disorder can play a crucial role in achieving broadband solar reflection and MIR thermal emission, by exploiting randomness in particle distribution, porous networks, or materials with intrinsically random structures.

Among the various strategies utilizing disorder, an effective way is randomly arranging dielectric or plasmonic particles within a host matrix to control emissivity and enhance scattering for effective thermal radiation within the atmospheric transparency window. An early study by Gentle and Smith [158] demonstrated that embedding phonon-resonant nanoparticles (e.g., SiC, SiO_2_) within IR-transparent polymers could yield highly selective IR emission confined to the atmospheric window, while maintaining solar transparency and material simplicity. Owing to their spectrally tuned absorption characteristics that avoid major atmospheric absorption bands, these nanocomposites achieve impressive cooling performance, with modeled stagnation temperatures reaching up to 25 °C below ambient and net radiative cooling powers exceeding 40–50 W m^−2^ under dry, clear night conditions. Another notable example is a glass-polymer hybrid metamaterial [81] designed by embedding randomly distributed SiO_2_ microspheres within a polymethylpentene (TPX) matrix, as shown in Figure 4A. The strong MIR absorption is enabled by Fröhlich resonance of SiO_2_ microspheres at 9.7 µm, while broadband scattering further enhances light–matter interaction. The resulting structure achieves high infrared emissivity (≥0.93) while reflecting over 96 % of solar radiation. Similarly, a silica–polymer metamaterial [159] achieves spectral decoupling of cooperative emissivity by balancing contributions from SiO_2_ microspheres and polymer phonon absorption. This structure benefits from both phonon–polariton interactions and scattering-induced optical path length enhancement, which results in a broadband, high emissivity profile suitable for radiative cooling. Additionally, a metafilm can also be made by integrating randomly distributed SiO_2_ microspheres within a polymer matrix, with a silver layer backing up to prevent thermal losses [160]. This configuration cools water to 10.6 °C below ambient temperature at noon under direct sunlight.

While randomly dispersed scatterers in polymeric matrices offer strong radiative cooling performance, another widely adopted approach is the use of radiative cooling paint, owing to its low cost [161], [162], [163], ease of application [164], [165], and scalability [166], [167]. A typical radiative cooling paint consists of pigment particles and resin [168]. Recent advancements in material selection and particle size optimization [169] have further enhanced cooling performance, making them a promising strategy for passive radiative cooling.

One type of radiative cooling paint contains high-refractive-index, highly scattering microparticles, typically single-component inorganic materials, that passively reflect sunlight and radiate heat. For example, barium sulfate (BaSO_4_), widely recognized for its high bandgap and excellent scattering properties, is used in ultrawhite paints capable of 98.1 % solar reflectance and 0.95 longwave IR emissivity [58]. Similarly, a magnesium oxide (MgO)-based coating [170] exhibits 95 % solar reflectance and 93 % IR emissivity. An SiO_2_-enhanced radiative cooling paint [171] also enhances longwave IR emission by embedding SiO_2_ particles into a polymethylpentene–acrylic resin matrix. To further enhance cooling performance, multimaterial microparticle composites combining SiO_2_ and aluminum oxide (Al_2_O_3_) have been studied [169]. These high-bandgap oxides, when integrated into a polymeric matrix, enable optimized scattering and strong thermal emissivity, achieving solar reflectance values up to 0.958. Alternatively, core–shell particles consisting of a core material coating with one or more shell material layers have been studied [155], as shown in Figure 4B, where the particle system was designed using SiO_2_ cores coated with willemite (Zn_2_SiO_4_) shells to enhance light scattering in the solar spectrum while exploiting IR phonon resonance for efficient thermal emission.

Beyond such pigment-based and core–shell particle approaches, some works also employ polymeric materials as the primary cooling component. Instead of relying solely on inorganic scattering pigments, these coatings integrate polymeric matrices with carefully selected IR-emitting functional groups. For instance, an aqueous-based, volatile organic compound-free radiative cooling paint [172] was formulated using polymeric emulsions combined with glass beads, achieving subambient cooling of up to 6 °C even in humid climates. Similarly, a scalable paint-format microparticle–polymer composite [163] combined Al_2_O_3_ and SiO_2_ microparticles in a polymer binder, achieving 94.1 % solar reflectance.

Another important advancement in radiative cooling paints involves mechanically processed materials, particularly those optimized through ball-milling techniques. This method is used to break down larger particles into smaller, more efficiently scattering sizes, thereby enhancing solar reflectance. As an example, a glass bubble-based radiative cooling paint [173] utilized ball-milled hollow glass microspheres to increase solar reflectivity from 93.3 % to 97.3 %, achieving a temperature drop of 3.5 °C at noon and up to 14.1 °C at night when combined with nanoporous polyethylene coverage. Similarly, a cost-effective radiative cooling paint [174] employed larger, milled glass bubbles, leading to a solar reflectivity improvement from 75.8 % to 95.8 %. Also, a zirconium dioxide (ZrO_2_)-based radiative cooling paint [57] utilized ball-milled ZrO_2_ microparticles to enhance both solar reflectance and mechanical durability, achieving an abrasion resistance of 5,310 m N mm^−3^, far exceeding commercial alternatives.

Paint-based coolers represent one of the most practical and scalable solutions for passive cooling applications, offering broad material compatibility, ease of application, and cost-effectiveness. Still, long-term durability and environmental safety, among other factors, must be addressed prior to widespread adoption.

While paint-based radiative cooling strategies achieve high reflectance and emissivity using pigments and polymer matrices, fiber-based materials offer an alternative approach by leveraging their unique morphologies for enhanced light scattering and thermal emission. A fiber is generally considered as a one-dimensional material characterized by a high aspect ratio where its length is significantly greater than its diameter, typically ranging from submicron to hundreds of micrometers [175]. Fibers can be natural [176], [177] or synthetic [178] and are characterized by their porous, interwoven, or aligned networks.

A widely studied class of fiber-based radiative cooling materials consists of synthetic fiber membranes, where polymeric or inorganic fibers are engineered to optimize optical and thermal properties. For instance, a silica fiber network [156], depicted in Figure 4C, was numerically optimized using a Monte Carlo method, demonstrating that fibers suspended in air can significantly enhance backscattering and IR emissivity. Multiple reflections in disordered emissive structures backed by metallic reflectors may induce parasitic absorption at the metal interface. However, scattering within the disordered layer reduces photon flux reaching the metal, mitigating this effect. Similarly, for incident solar radiation, increased optical path length due to scattering lowers direct metal interaction, while multiple reflections near the metal/dielectric boundary may slightly increase absorption. Overall, these opposing effects largely compensate, resulting in minimal impact on solar reflectance. Furthermore, a ceramic fiber-based radiative cooling paper [179] was designed for personal thermal management, achieving a 12.9 °C reduction compared to conventional cotton fabrics. Electrospun polymer composites have also been explored; a nanoporous fiber-based radiative cooling textile [180], integrating core–shell silica microspheres, achieved 98.8 % solar reflectivity and 97 % IR emissivity, reducing skin temperature by 7.1 °C under direct sunlight.

Beyond synthetic fiber networks, hybrid fiber membranes incorporating metal oxides or composite materials have been developed to further enhance cooling efficiency and durability. A polystyrene–metal oxide fiber composite [181] demonstrated nearly 100 % visible and near-IR reflectivity, achieving 22.3 °C subambient cooling, outperforming many conventional cooling coatings. Similarly, a waterproof and breathable radiative cooling membrane [182] was fabricated using nanoarchitectured fiber mesh, providing superior air permeability while reducing temperature by 7.9 °C compared to commercial cotton textiles.

In addition to synthetic and composite fiber membranes, naturally derived cellulose fibers present an eco-friendly alternative with intrinsic optical and thermal properties that make them well-suited for radiative cooling. Cellulose is a naturally occurring fiber composed of long-chain polysaccharides that form the primary structural component of plant cell walls [183]. Unlike synthetic polymer fibers, which require chemical modifications to achieve optimal emissivity, the intrinsic structure and surface chemistry of cellulose naturally enable high solar reflectivity and MIR radiation, allowing it to passively cool surfaces without additional processing.

A primary example of this is the engineering of delignified wood, where lignin is removed to expose and densify cellulose nanofibers, resulting in radiative cooling performance alongside increased mechanical strength. A densified cooling wood [50] was fabricated through chemical delignification and mechanical pressing, achieving broadband solar reflectivity and strong MIR emissivity, enabling continuous cooling both day and night. When applied as a building material, this cooling wood was projected to reduce energy consumption by 20–60 %, particularly in hot and dry climates.

Other advancements in cellulose-based radiative cooling materials have explored biodegradable, high-performance fiber membranes and engineered cellulose composites for cooling applications. A biodegradable silk fibroin–polylactic acid (PLA) fiber membrane [157], depicted in Figure 4D, achieved 96.1 % solar reflectance and 95.4 % IR emittance, leading to an average subambient cooling effect of 6 °C, demonstrating an eco-friendly, energy-free cooling strategy. Similarly, a cellulose-based hybrid structural material [184] integrated inorganic microspheres into a 3D bulk fiber network, enabling 24-h cooling, reducing temperatures by up to 8 °C, while offering mechanical strength surpassing natural wood.

Disordered media excel at broadband scattering and are often simpler to fabricate than precisely patterned crystals or cavities. However, randomness can also mean less spectral finesse: performance is broad but may not be as sharp as in carefully tuned resonant systems. Additionally, achieving angular robustness might require carefully managing the thickness and density of scatterers.

**Figure 5:**
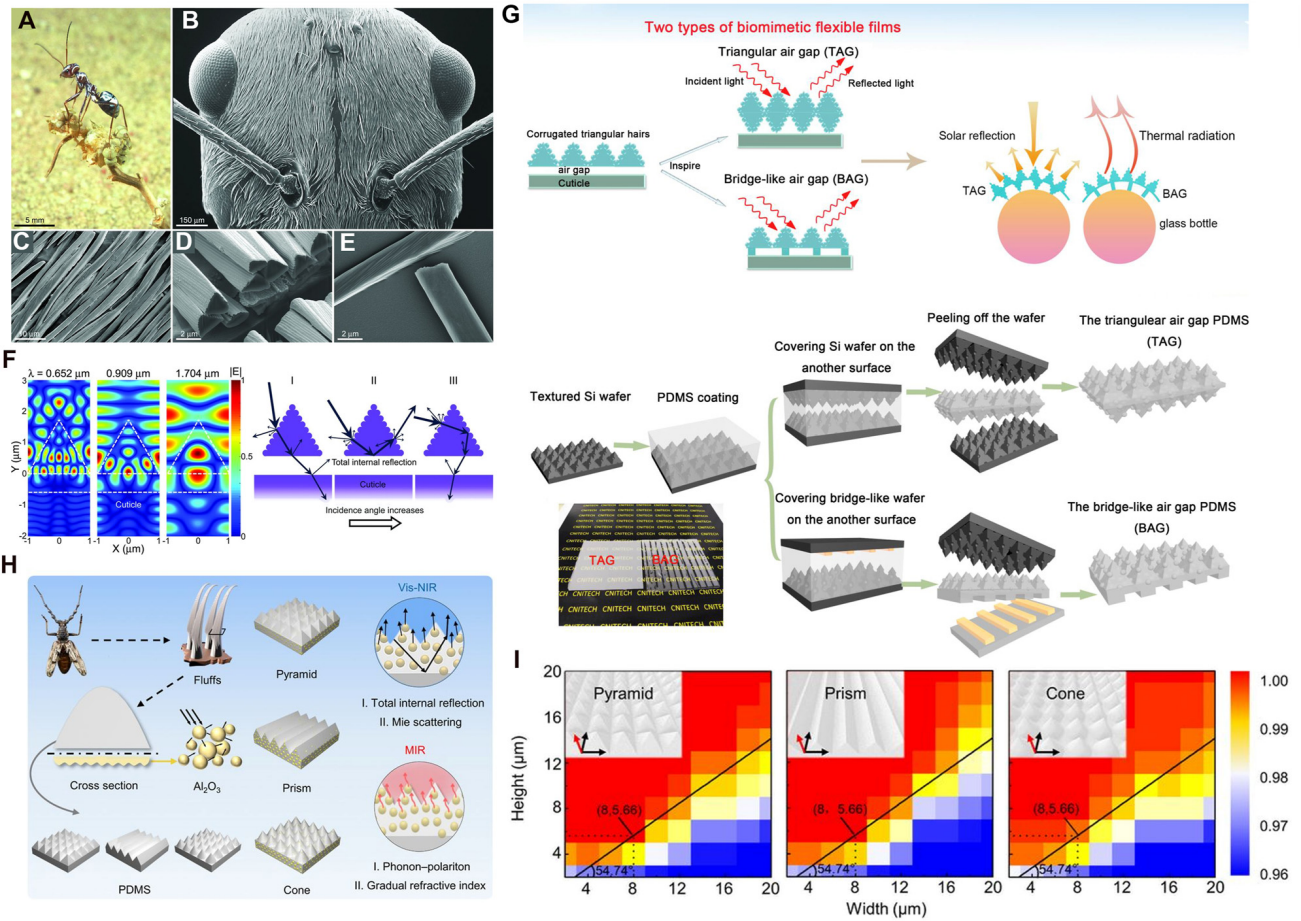
Example works incorporating biomimicry for radiative cooling. (A)–(F) The thermoregulatory properties of Saharan silver ants (*Cataglyphis bombycina*) are investigated, demonstrating how their unique triangular hair structures enhance solar reflectivity through Mie scattering and total internal reflection while simultaneously increasing MIR emissivity for radiative heat dissipation. From [185]. Reprinted with permission from AAAS. (A) Shows a silver ant in its natural habitat, while (B)–(E) present SEM images detailing the ant’s hair structure and cross section. (F) Illustrates optical simulations revealing how the hair geometry contributes to efficient light scattering and heat dissipation. (G) A flexible biomimetic photonic film inspired by the hair-like structures of Saharan silver ants is fabricated, designed to enhance solar reflection and infrared emission for efficient passive cooling [186]. Reproduced with permission, Copyright 2020 Elsevier. (H)–(I) Develop a biologically inspired flexible photonic film for passive radiative cooling, mimicking the dual-scale microstructure of longicorn beetle fluffs. Adapted with permission from [109]. (H) Shows the hierarchical photonic structure inspired by beetle fluffs, and (I) presents an optimization analysis of different microstructures, comparing pyramidal, prismatic, and conical geometries for maximizing radiative cooling efficiency.

### 3.2 Biomimetic strategies

Biomimetic strategies for radiative cooling take inspiration from natural systems that have evolved to regulate temperature efficiently across diverse environments. Many organisms have evolved specialized surface features that manage heat efficiently by enhancing solar reflectivity and thermal radiation. For instance, *Cerambycini latreille* achieves radiative cooling via densely packed, tapered hair-like structures that increase IR emissivity while reflecting sunlight [187]. Meanwhile, *Morimus asper funereus* utilizes a hierarchical arrangement of blackbody-like cavities, microlens, and hyperuniform photonic structures to enhance IR absorption and emission [188]. These biologically inspired mechanisms offer valuable design methodologies for developing radiative cooling materials with tailored optical properties to achieve efficient heat management.

Saharan silver ants (*Cataglyphis bombycina*), shown in Figure 5A, withstand extreme desert temperatures using triangular-shaped hairs shown in Figure 5B–E that reflect sunlight in the visible and NIR spectrum while emitting thermal radiation in the MIR atmospheric window [185], [186], as visualized in Figure 5F. This dual functionality minimizes solar heat absorption and enhances radiative heat dissipation, enabling the ants to maintain body temperatures below critical thresholds.

Building on this natural adaptation, recent studies have developed biomimetic radiative cooling materials that replicate the thermoregulatory properties of this species, an example shown in Figure 5G. For instance, inspired by the triangular hairs of the ants, prismatic microstructures have been incorporated into a polydimethylsiloxane (PDMS)-based polymer, exploiting gradient refractive index effects to enhance solar reflection and infrared emission, achieving a net cooling of 6.2 °C in field tests [189]. The hierarchical arrangement of ant hairs has also been replicated in a flexible photonic architecture, optimizing light scattering and thermal radiation for photovoltaic applications, thereby improving energy efficiency and reduces thermal losses [47]. Numerical studies on silver ant-inspired micro–nano structures show that varying the air gap and tip height in biomimetic pyramid, tetrahedral, and triangular prism geometries can significantly enhance solar reflectivity while maximizing MIR emissivity [190].

While Saharan silver ants are well known for their radiative cooling mechanisms, other insects also exhibit passive cooling strategies that have inspired advanced radiative cooling structures. For instance, longicorn beetles (*Neocerambyx gigas*) possess dual-scale fluffs with a triangular cross section, as depicted in Figure 5H, that enable total internal reflection and Mie scattering, minimizing solar absorption. This functionality has been replicated by fabricating a micropyramid-arrayed polymer matrix embedded with ceramic particles, achieving 95 % solar reflectance, over 96 % MIR emissivity, and a passive cooling effect of 5.1 °C under direct sunlight [109], with the optimization analysis depicted in Figure 5I. Similarly, *Morpho didius* butterflies use nanostructured ridges to manipulate light via interference and broadband reflection, a principle adapted to near-field radiative cooling through SiC-based palm tree-like metamaterial emitters [191]. By tuning the geometric and optical properties, the emitter structures enhance radiative heat transfer within the infrared atmospheric window, with numerical simulations showing that a 90° rotation of the emitter further maximizes cooling efficiency.

Extending beyond insects, nature provides a much broader range of biological systems that exhibit passive thermal regulation, spanning from skeletal structures, plant-based adaptive mechanisms, and even planetary thermal regulation, to enhance radiative cooling and energy efficiency. As an example, inspired by the high mechanical strength and thermoregulatory function of porous bone structures, a cellulose-based bio-film with embedded hydroxyapatite ultra-long nanowires [192] is developed. This bio-inspired design mimics the light-scattering properties of skeletal structures, achieving over 95.59 % solar reflectivity and 96.73 % MIR emissivity. Also, the self-folding leaves of *Mimosa pudica* plant, which contract upon touch or temperature fluctuations, served as the inspiration for a temperature-adaptive module [193]. This system consists of a Janus bilayer structure with photothermal nanoparticles that expand and contract in response to temperature shifts. This dual-mode passive thermal regulation provides an all-season, energy-efficient solution for buildings, reducing both cooling and heating demands. Furthermore, the Earth’s atmosphere has also been mimicked by [194] by developing a gradient Reduced Graphene Oxide (RGO) aerogel-based bilayer phase change composite. Just as Earth’s atmosphere absorbs, stores, and re-emits heat to stabilize temperatures, this material integrates solar-thermal energy conversion and latent heat storage, providing dynamic self-adaptive thermal regulation. The bottom heavily reduced RGO layer converts solar energy into heat, while the top phase-change material layer stores and releases heat as needed. This enables the material to retain warmth in frigid environments while also preventing overheating at high temperatures.

The biomimetic approach provides ready-made structural solutions for radiative cooling, reducing the need for intensive designing while ensuring high cooling efficiency. By directly adopting naturally occurring surface patterns, passive cooling with minimal material engineering can be realized. However, these naturally existing structures are not necessarily optimized for maximum radiative cooling efficiency. Additionally, replicating them at scale using existing fabrication methods can be challenging. Furthermore, in many cases, the radiative cooling performance arises not just from the material itself but from its naturally occurring structural arrangement. Since these structures are often composed of organic materials, directly replicating both the form and function using conventional fabrication techniques may not always be feasible.

### 3.3 Computational optimization

Designing high-performance radiative cooling structures often involves balancing multiple, interdependent parameters such as material choice, layer thickness, and geometric features across large, complex search spaces. While trial-and-error or purely analytical approaches can provide insights, computational optimization computational optimization systematically navigates these complex, high-dimensional design spaces, automating the discovery of near-optimal solutions that traditional empirical or intuition-driven approaches might overlook. In particular, these computational methods can reveal structures whose complex and nonintuitive geometries would be challenging to discover through intuition or analytical methods alone. In essence, computational optimization methods do not replace physical insight but augment it, allowing for systematic exploration of a far broader range of possibilities and rigorous co-optimization of multiple design criteria simultaneously. Two broad categories of algorithms, namely, global search and gradient-based/topology optimization, are widely used in radiative cooling research.

**Figure 6:**
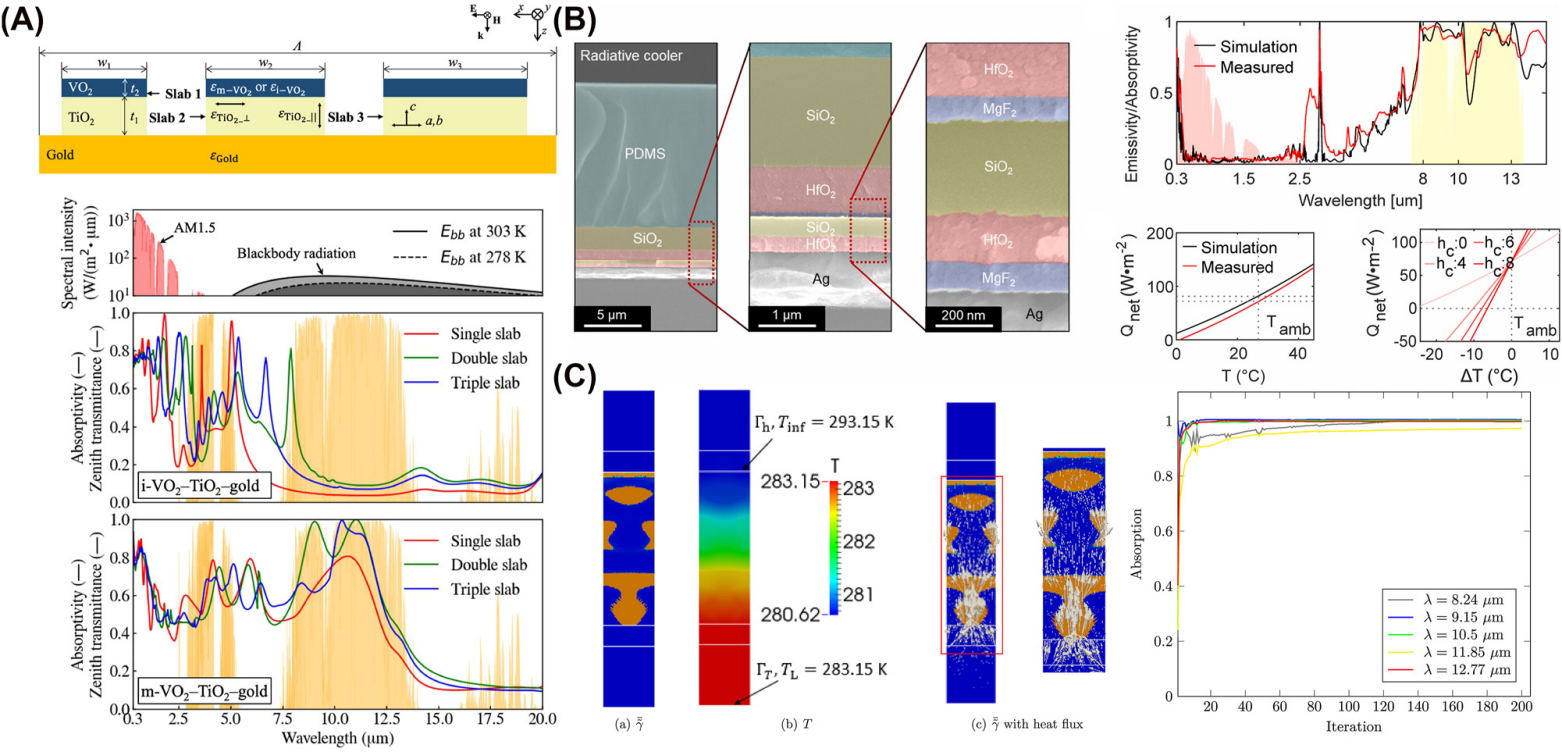
Works incorporating computational optimization methods for radiative cooling. (A) By coupling Bayesian optimization with FDTD simulations, periodic multilayered slabs containing VO_2_ were designed to passively switch between solar absorption and IR emission [195]. This approach identifies precise geometric parameters to maximize energy savings through tunable optical resonance and a thermochromic metal–insulator transition. Reproduced with permission, Copyright 2024 Elsevier. (B) A carefully configured multilayer stack, discovered via a genetic algorithm, achieves highly suppressed solar absorption and enhanced thermal radiation [196]. Its selective emission properties enable subambient cooling under direct sunlight without compromising efficiency. Licensed under CC BY 4.0. (C) Topology optimization distributes reflective material throughout an enclosure, tailoring boundary emissivities for controlled radiative heat transfer [197]. The resulting method systematically minimizes or maximizes net heat flux or temperature fields across targeted surfaces. Reproduced with permission, Copyright 2015 Elsevier.

#### 3.3.1 Global optimization algorithms

Many global search methods typically rely on iterative improvement guided by performance evaluations, which may come from electromagnetic solvers incorporating rigorous coupled wave analysis (RCWA) or finite-difference time-domain (FDTD) methods. In radiative cooling, global algorithms are especially valuable for handling highly nonlinear objectives such as maximizing solar reflectance while selectively enhancing MIR emissivity.

Among the various global optimization algorithms, Bayesian optimization stands out as one of the effective approaches for tackling radiative cooling problems. Bayesian optimization is a probabilistic global search optimization algorithm particularly well-suited for expensive black-box optimization problems [198], [199], making it useful for radiative cooling applications where the gradients of the objective function are difficult or impossible to compute. It operates by constructing a surrogate model, typically a Gaussian process [200], to approximate the objective function while maintaining an uncertainty estimate. The process starts with an initial sampling of designs, evaluated using computationally intensive simulations (e.g., RCWA or FDTD). These data train the surrogate model, and an acquisition function (such as expected improvement or upper confidence bound) then balances exploration and exploitation to select the next design for evaluation, iterating until convergence.

A notable application of Bayesian optimization is in the design of highly selective thermal emitters [201]. Here, the optimization algorithm was integrated with RCWA simulations to efficiently explore multilayer and grating-based structures. The Bayesian approach significantly reduced the number of simulations required, achieving an optimized spectral emissivity profile with fewer than 1 % of the total candidate structures evaluated. In the design of color-compatible radiative cooling structures [202], Bayesian optimization was used to optimize multilayered photonic structures that maintain aesthetic color properties while maximizing radiative cooling performance. The optimization guided the selection of candidate designs, ensuring that each iteration improved both optical aesthetics and thermal emission characteristics. Hollow microcavities for enhanced directional thermal emission [203] are also optimized by utilizing Bayesian optimization. The optimization iteratively refined structural parameters, including cavity diameter, height, and SiO_2_/AlO_X_ shell thicknesses, to maximize emissivity contrast and angular selectivity at key infrared wavelengths (here, 8, 9.1, 10.9, and 12 µm). In the optimization of multilayered slabs for passive absorptivity control [195], depicted in Figure 6A, Bayesian optimization was applied to design periodic VO_2_-TiO_2_ structures that regulate sunlight absorption and radiative cooling. With a Gaussian process surrogate model, the optimization efficiently identified slab geometries that enhance short-range surface plasmon polariton modes, improving spectral emissivity profiles.

Because each new design is selected based on both the surrogate model’s predicted performance and its uncertainty, Bayesian optimization helps reduce the risk of getting stuck in local optima [204]. However, Bayesian optimization struggles with high-dimensional design spaces, where the computational complexity scales rapidly [205].

Recognizing this issue, an adversarial autoencoder-assisted Bayesian optimization scheme [206] was proposed to overcome the challenges of high-dimensional design spaces in the development of narrowband thermal metamaterials. The adversarial autoencoder used in the framework first reduces the complexity of the design problem by mapping the high-dimensional space into a compact, low-dimensional latent space. This reduction enables the Bayesian optimization algorithm to perform efficient global searches within this simplified space, drastically reducing the computational cost by requiring direct evaluation of less than 0.001 % of the total candidate structures. Nonetheless, while this approach significantly reduces the computational burden, it is not without challenges. For sufficiently complex problems, even with dimensionality reduction, the optimization process may still require extensive computation to converge to an optimal solution.

Another widely used global optimization algorithm is the genetic algorithm (GA). GA is a global optimization algorithm drawn from the concepts of genetics and natural selection found in biological systems [207]. GA explores a population of potential solutions, iteratively evolving them through selection, crossover, and mutation while simultaneously evaluating multiple solutions in parallel to explore the design space efficiently. This process enables the discovery of optimized material compositions, structural geometries, and layer thicknesses for radiative cooling applications, where objective functions are often computationally expensive and highly nonlinear.

As an example, GA has been applied to optimize the thicknesses and material compositions of multilayer thin films [196], shown in Figure 6B, to enhance passive radiative cooling performance for both daytime and nighttime. Their approach explored a large parameter space efficiently, using cooling power as the FoM and iteratively refining candidate structures through crossover and mutation. A multifunctional emitter capable of switching between IR stealth, thermal imaging, and radiative cooling [208] was also optimized with GA by precisely tuning the thicknesses of Mo/GST multilayers. Their fitness function was based on absorptivity contrast between the dual-band atmospheric window (MIR and LIR) and the nonatmospheric window, allowing the GA to evolve structures that achieved dynamic spectral switching. GA can be integrated with other optimization algorithms such as simulated annealing to optimize multilayer thin films [209] for passive radiative cooling, constraining layer thicknesses and material choices within a predefined library. The study identified 30 optimized structures suited for daytime cooling, nighttime cooling, and water-soluble materials, balancing radiative, conductive, and convective heat transfer contributions. Other exemplary works incorporating GA for radiative cooling can be found in [210].

Despite the effectiveness of GAs, a primary concern when using the algorithm is premature convergence, where the algorithm may settle into a local optimum before exploring the full design space [211]. Also, GAs can be computationally expensive in cases where large amounts of design variables need to be tuned, particularly in large-scale design problems [212]. Nonetheless, when these challenges are addressed properly, GAs can serve as an efficient method in optimization.

There is also a family of optimization algorithms inspired by collective behaviors in nature, such as bird flocking or insect colonies, referred to as swarm intelligence algorithms [213], [214]. Each algorithm in this group models solutions as “agents” (or “particles”) that communicate or learn from one another, iteratively gravitating toward promising regions of the design space.

One of the most common swarm intelligence algorithms, particle swarm optimization (PSO) [215], optimizes radiative cooling designs by treating each candidate solution as a particle moving through the parameter space, where its position represents a specific set of values for design variables such as layer thickness, material composition, or pattern geometry. Each particle’s velocity dictates its movement, and the swarm initializes with random positions and velocities. The objective function, such as net cooling power, is evaluated at each position, tracking the best solutions found by individual particles and the entire swarm. Over iterations, particle velocities are updated through three components: inertia, which maintains part of the previous velocity for smoother exploration; cognition, which directs the particle toward its best-known solution; and social influence, which steers it toward the global best. These adjustments balance exploration and exploitation, refining the particle’s position to improve the objective function evaluations.

An example implementation of PSO is the optimization of the structural parameters of a selective multilayered periodic grating radiator for radiative cooling [216]. The algorithm was utilized to find optimal values for the grating period, duty cycle, and grating height to maximize the cooling power. In this work, the objective function is formulated to minimize the absorbance difference between the target emissivity and the radiative emissivity of the designed structure, so that the optimized structure closely follows the desired emissivity and reflectivity profiles within the specified wavelength ranges. PSO has also been used in the optimization of the stacking sequence and thickness of an inorganic multilayer structure for daytime radiative cooling [99].

In addition to PSO, a recent swarm intelligence method, sparrow search algorithm (SSA) [217], has also been applied to radiative cooling. SSA has been utilized in [116] for thermal management, particularly in optimizing film cooling by tuning the geometric and aerodynamic parameters of semi-sphere vortex generators.

Because swarm intelligence algorithms do not require explicit gradients, they can efficiently tackle radiative cooling problems involving irregular geometries or nonlinear responses. However, these algorithms are often susceptible to premature convergence [218]. Also, their performance heavily depends on the balance between exploration and exploitation [214]. Thus, improving diversity mechanisms [219], [220], [221] and careful parameter control may be necessary.

While swarm intelligence algorithms excel in exploring complex, high-dimensional design spaces through distributed agent-based searches, another widely used global optimization approach, simulated annealing (SA), draws its inspiration from the annealing process used in metallurgy. SA is a global optimization method that explores the radiative cooling design space by iteratively testing new configurations and probabilistically accepting less favorable changes to escape local optima [222]. The algorithm’s exploration is governed by a temperature parameter, which begins high to promote broad searching and gradually decreases to refine solutions, balancing global exploration with local optimization.

In SA, the algorithm begins with an initial design, such as a multilayer stack or photonic crystal configuration, and evaluates its objective function, typically representing net cooling power or emissivity efficiency. A neighboring design is generated through small, random modifications. If the new design improves performance, it is accepted; otherwise, it may still be accepted with a probability that decreases with the performance drop and current temperature. This probabilistic acceptance helps escape local optima and explore a broader design space. As the algorithm progresses, the temperature is lowered following a cooling schedule, reducing the likelihood of accepting worse solutions and shifting from exploration to exploitation. The process continues until a minimal temperature threshold or stopping condition is reached, yielding the best-found solution.

An example application of SA is in the optimization of a metallodielectric multilayer system for radiative cooling [223]. By refining the number and thickness of alternating SiO_2_ and PMMA layers on an Ag mirror, they maximized net cooling power while accounting for radiative emission, atmospheric absorption, solar absorption, and convective losses. SA has also been applied in the inverse design of spectrally selective pigmented coatings, optimizing pigment size, volume fraction, and thickness to achieve ideal spectral properties for solar thermal applications [224]. By tuning pigment radius, volume fraction, and coating thickness, the algorithm minimized deviations from an ideal spectral emittance profile while considering net radiant heat flux. To improve accuracy, the optimization incorporated a unified radiative transfer model that combined Lorenz–Mie theory, extended Hartel theory, and the transfer matrix method, ensuring a physically accurate solution while helping the SA algorithm escape local minima.

Because simulated annealing does not rely on gradients or assumptions of smoothness, it suits radiative cooling problems with complex simulation requirements or discontinuous objective landscapes. The main challenge lies in properly choosing the initial temperature, cooling schedule, and neighborhood generation scheme. These factors significantly influence the algorithm’s capacity to traverse broad swaths of parameter space early on while still honing in on a near-optimal design toward the end. When appropriately tuned, simulated annealing provides a viable balance between global exploration and local refinement, enabling the discovery of innovative radiative cooling structures that might be elusive under purely deterministic approaches.

#### 3.3.2 Topology optimization

Topology optimization is a powerful method for arranging materials and structures to achieve efficient radiative cooling. Rather than tuning only a few layer thicknesses or repeating units, it systematically distributes material throughout a design space to maximize (or minimize) a chosen objective such as boosting infrared emission or reducing solar absorption. Using gradient-based algorithms, topology optimization can handle large numbers of design parameters at once. This high degree of freedom lets it discover unconventional structures that conventional design approaches might miss. The trade-off is that the resulting geometries can be complex, sometimes requiring specialized fabrication or constraints to ensure practicality. Nonetheless, topology optimization remains an attractive route for discovering innovative, high-performance cooling solutions that precisely manipulate radiation in the solar and thermal infrared bands.

One of the most computationally efficient methods for calculating the gradients numerically in electromagnetic and thermal systems is the adjoint method, the theoretical background described in detail in [225], [226]. The adjoint method enables calculation of gradient with only two simulations: one forward and one backward (adjoint), regardless of the number of design variables. This makes it an indispensable tool for optimizing various radiative cooling structures, serving as the foundation for more advanced techniques.

Given an objective function *F* that depends on an electromagnetic field distribution **E** and **H**, design parameters *α*, and computational domain Ω, we express it as:

(3) $$F\;\left(E,H,\alpha \right)=\underset{\Omega }{\int }f\;\left(E,H,\alpha \right)dV$$

The electromagnetic fields are governed by Maxwell’s equations. To compute the gradient $\frac{dF}{d\alpha }$ , we differentiate implicitly using the chain rule:

(4) $$\frac{dF}{d\alpha }=\frac{\partial F}{\partial \alpha }+\frac{\partial F}{\partial E}\frac{\partial E}{d\alpha }+\frac{\partial F}{\partial H}\frac{\partial H}{d\alpha }$$

Letting $D=\epsilon \;\left(\alpha \right)E$ and $B=\mu \;\left(\alpha \right)H$ , since the fields are constrained by Maxwell’s equations, we introduce adjoint variables E* and H* that satisfy the adjoint Maxwell equations:

(5) $$\nabla \times H^{*}-\frac{\partial D^{*}}{\partial t}=-\frac{\partial F}{\partial E}$$

(6) $$\nabla \times E^{*}+\frac{\partial B^{*}}{\partial t}=-\frac{\partial F}{\partial H}$$

By solving the adjoint problem, the gradient of the objective function pertaining to all design parameters can be obtained by:

(7) $$\frac{dF}{d\alpha }=\underset{\Omega }{\int }\left(E^{*}\cdot \frac{\partial D}{\partial \alpha }+H^{*}\cdot \frac{\partial B}{\partial \alpha }\right)dV$$

This shows that the adjoint method efficiently provides gradients by the reciprocity principles, eliminating the need for repeated forward simulations.

However, there are situations where the adjoint method becomes impractical, particularly when the objective function exhibits strong nonlinearity, discontinuities or dependencies in intermediate computational steps that are difficult to differentiate analytically. In this case, the adjoint source formulation can become challenging [227]. In such cases, automatic differentiation [228] offers an alternative approach by systematically differentiating through the computational graph of the objective function, ensuring gradients without symbolic differentiation or finite differences.

Topology optimization has been effectively employed to design radiative cooling structures, enabling the systematic distribution of materials to optimize spectral selectivity and thermal emission control. By discretizing the design space into computational elements, this method significantly enhances the degrees of freedom for optimizing photonic and thermophotonic structures [227].

Adjoint-based topology optimization has been widely used for optimizing material distributions in broadband thermal emitters and thermophotonic structures. For instance, adjoint-based topology optimization was applied to structure the distribution of SiO_2_ within a TPX matrix [117], incorporating finite element analysis to solve Maxwell’s equations and heat conduction problems while enforcing binary material distributions using SIMP interpolation and boundary slope constraints. Alternatively, the method of moving asymptotes (MMA) [229] was also employed to optimize boundary reflectivities within radiative enclosures [197], the analysis shown in Figure 6C. MMA was used to optimize the distribution of reflective materials inside diffuse-gray enclosures by iteratively adjusting design variables through local convex approximations, ensuring stable and efficient convergence. MMA adaptively modifies upper and lower asymptotes for each variable, preventing overly aggressive or excessively small updates that could hinder optimization progress. The optimization framework was coupled with the net-radiation method to model radiative heat transfer, iteratively updating boundary reflectivities based on computed gradients.

While adjoint-based methods leverage efficient gradient computation via adjoint sensitivity analysis, MMA relies on numerical sensitivity evaluations, making it particularly useful when adjoint formulations become challenging due to complex governing equations or boundary conditions. Although MMA may be computationally intensive for large-scale problems, its flexibility in handling constraints and ensuring stable convergence makes it a valuable alternative for optimizing thermal radiation systems.

Topology optimization offers substantial design freedom, enabling complex and highly efficient structures. However, it often generates intricate geometries that challenge the capabilities of conventional fabrication techniques. The optimization process does not inherently account for manufacturing constraints, leading to designs that, while theoretically optimal in terms of performance, may be irregular, impractical or even impossible to produce using existing fabrication methods. To address this, various manufacturing constraints have been incorporated. For instance, SIMP-based interpolation was used to enforce discrete material distributions, while boundary slope constraints regulated material transitions, ensuring feasibility in layer-wise deposition techniques [117]. For thin-film coatings, a penalization-based continuation strategy was applied to optimize boundary reflectivities, systematically removing intermediate values to enhance manufacturability [197].

#### 3.3.3 Needle optimization

Needle optimization [230] is a specialized gradient-based optimization method that refines structures by making minimal yet highly impactful perturbations in the design space. Unlike global optimization techniques that modify large-scale parameters, it modifies a system by inserting thin-layered perturbations, often referred to as “needles,” at specific locations where they are most effective. This technique allows for fine control over a design, making it particularly useful for problems where large-scale changes could disrupt functionality.

The algorithm operates iteratively, starting with a baseline structure, which is often a multilayered or periodic material configuration. At each iteration, the system evaluates potential modifications by introducing small perturbations at key locations. These perturbations, which could involve adding new layers, adjusting refractive indices, or modifying thicknesses, are selected based on sensitivity analysis to maximize improvements in the objective function. Once a perturbation is introduced, the system recalculates the objective function to assess the impact of the change. If the modification leads to an improvement, it is retained, and the process continues with further refinements.

A notable example of needle optimization’s effectiveness in radiative cooling is its application in the design of multilayered photonic emitters for daytime passive cooling. In the work by Mohammed et al. [231], needle optimization was utilized to refine the structure of a thin-film emitter, optimizing material layer thickness and composition to enhance emissivity in the 8–13 µm range while maintaining high solar reflectivity. The process began with an initial four-layer configuration, which was then iteratively improved through needle perturbations to achieve a final ten-layer design with superior cooling performance. The work by Raman et al. [80] also implements the needle optimization method to determine and optimize the number of layers and layer thicknesses of the photonic radiative cooler.

One of the primary advantages of needle optimization is its high precision, which enables localized improvements without drastic structural modifications. It is also computationally efficient, requiring fewer iterations compared to brute-force parameter sweeps. Moreover, it ensures smooth convergence by focusing on small perturbations, thereby avoiding instability in complex design spaces. However, it has limitations, including sensitivity to local optima, which means that it may converge to suboptimal solutions. Additionally, when applied to large-scale problems, the evaluation of gradients may become computationally expensive.

**Figure 7:**
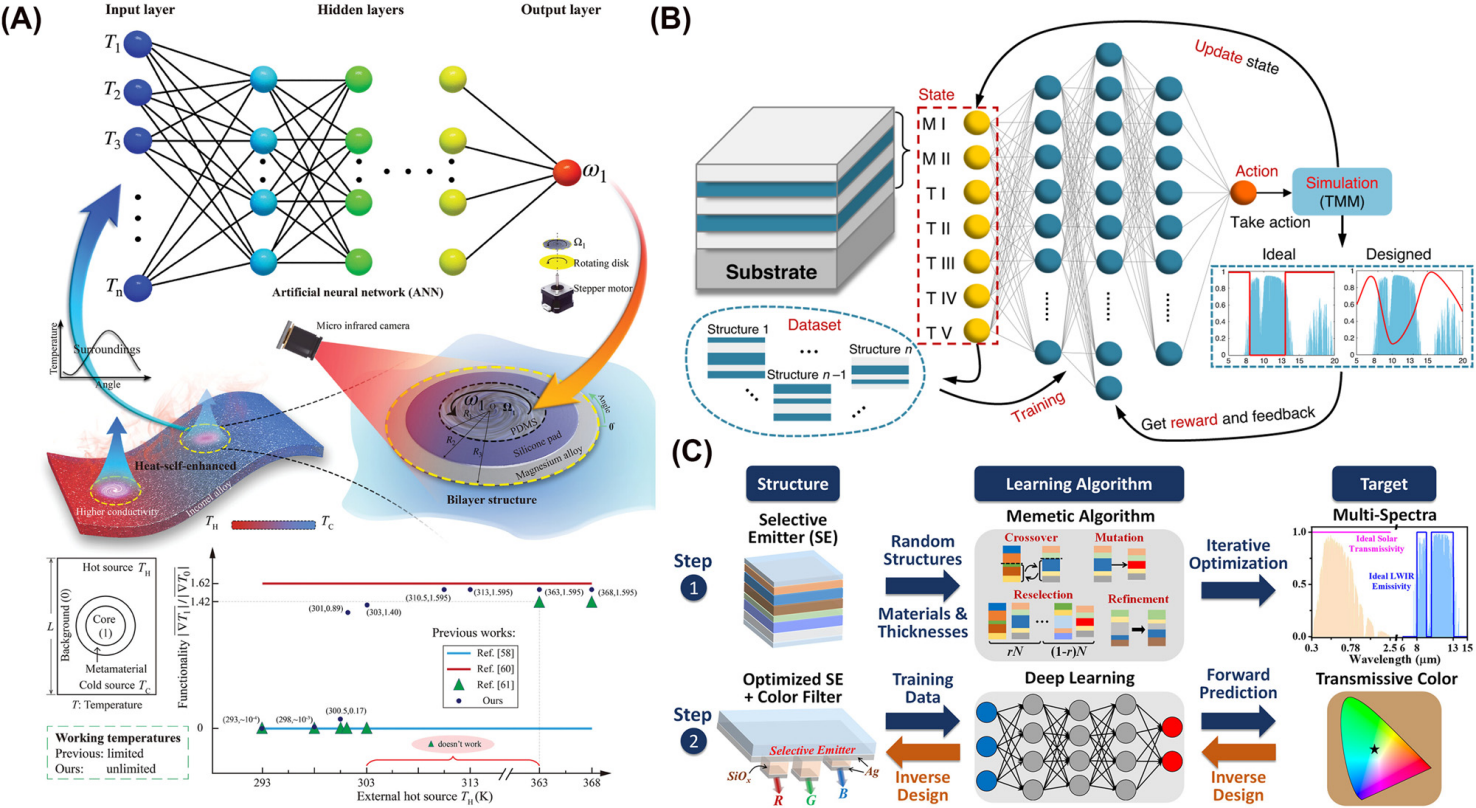
Works incorporating data-driven methods for radiative cooling. (A) By embedding a pretrained deep-learning network into thermal diffusion metamaterials, these active structures automatically sense and adapt to changing thermal fields. Their rotating core layer markedly alters effective conductivity, delivering robust thermal management even in complex environments. Reprinted with permission from [232]. (B) A general emissivity-engineering framework integrates a deep reinforcement learning algorithm with multilayer design, autonomously selecting both materials and geometry for application-specific emissivity spectra. This pipeline efficiently delivers wavelength-selective emitters that excel in diverse tasks such as thermal camouflage, radiative cooling, and infrared sensing [233]. Licensed under CC BY 4.0. (C) A data-driven inverse design method instantly predicts multilayer radiative cooling films with prescribed transmissive colors for aesthetic integration in real-time. By combining a memetic algorithm for emitter optimization and a tandem neural network for color generation, the approach tailors solar reflection, thermal emissivity, and visible transmittance simultaneously [234]. Licensed under CC BY 4.0.

### 3.4 Data-driven methods

In the design of radiative cooling materials, artificial intelligence (AI), driven by data, has emerged as a transformative approach that surpasses conventional trial-and-error methodologies. Using machine learning models and various advanced frameworks, data-driven methods uncover intricate connections between structural parameters and optical characteristics or other intermediate values, facilitating swift design exploration and optimization. Crucially, data-driven methods complement physical understanding by revealing nonobvious relationships within design data, enabling the rapid prediction of high-performing structures that might elude conventional approaches. Unlike traditional simulations that rely on computationally expensive physics-based solvers, these methods accelerate the search for optimal configurations, useful in both forward modeling and inverse design tasks. Additionally, techniques such as tandem neural networks refine inverse design by ensuring that predicted structures yield physically meaningful optical responses, while hybrid approaches that integrate machine learning with other independent frameworks or optimization algorithms further enhance material discovery.

#### 3.4.1 Traditional neural networks

A traditional neural network is a class of AI models inspired by the human nervous system, created to identify patterns and connections within data. They are comprised of interconnected layers of neurons that process input data through weighted connections, enabling tasks such as classification, regression, and optimization. These models have been widely employed in scientific and engineering applications, including radiative cooling design.

Artificial neural networks (ANNs), a subclass of neural networks, have been extensively applied in radiative cooling design to predict the spectral response of various cooling structures. ANNs consist of multiple layers, including hidden layers, which enable them to capture intricate patterns within data. They have been successfully employed in predicting spectral emissivity, optimizing multilayer structures, and identifying novel radiative cooling materials. For instance, one study incorporated a fully connected ANN into an active metamaterial system that autonomously adjusts thermal transport properties in response to ambient temperature variations, thereby enhancing the performance of thermal signal modulators and thermoelectric generators, as outlined in Figure 7A [232]. In another work, ANN models were employed to optimize the infrared emissivity of radiative cooling windows by balancing broadband and selective emissivity in response to different climatic conditions and building configurations [235]. In a separate effort, a study utilized an ANN for the inverse design of ultra-narrowband selective thermal emitters, where the network rapidly mapped design parameters to achieve high spectral selectivity, with Q-factors as high as 109.2, and omnidirectional emission [236]. By training on a large dataset of over 200,000 samples, the model efficiently predicted the optimal multilayer configurations, enabling the fabrication of emitters with finely tuned spectral responses. Meanwhile, another study demonstrated an ANN-based framework to model various radiative heat transfer phenomena, ranging from near-field interactions in multilayer hyperbolic metamaterials to passive cooling in photonic crystal slabs, providing fast surrogate modeling for efficient inverse design [237]. Further exploiting ANN’s potential, one work applied it to optimize the design of ultra-narrowband thermal emitters, where the model predicted geometric and material parameters with remarkable accuracy, achieving a mean squared error below 0.006 [238].

Deep neural networks (DNNs), a deeper extension of ANNs with more than two hidden layers, offer superior representational capacity and have been instrumental in refining the design of radiative cooling materials. One study utilized a DNN-based framework to optimize nanoparticle-embedded polyethylene films for radiative cooling, where the model determined the optimal volume ratios, particle size distributions, and optical constants of oxide/nitride nanoparticle composites to achieve target spectral emissivity [239]. By integrating the transfer matrix method and effective medium theory, the trained DNN enabled direct inverse design, facilitating the rapid identification of materials with tailored transparency and longwave IR emission. Another work introduced machine learning to enhance the design of biomimetic radiative cooling metamaterials, specifically inspired by *Batocera lineolata* forewing structures, using SiO_2_-based metasurfaces [240]. A deep learning model trained to predict spectral responses revealed that truncated cone arrays exhibited high emissivity of 0.985 and high structural robustness. The model effectively guided inverse design by mapping spectral characteristics to geometric parameters, and a generative model was used to expand the inverse design space, significantly reducing computational costs compared to conventional optimization methods.

Neural networks, including ANNs and DNNs, offer significant advantages in radiative cooling design by approximating complex nonlinear functions to efficiently predict optical properties, material performance, and thermal behavior. Their flexibility enables them to capture intricate data relationships, making them highly effective for predictive modeling. However, these models also face challenges, particularly their typical reliance on large, well-labeled datasets [241]. Overfitting is a common issue, where models become excessively complex and capture random fluctuations rather than true patterns, leading to poor generalization. This necessitates techniques such as dropout [242], [243] or batch normalization [244] to improve model robustness. Conversely, underfitting can occur when a model lacks sufficient complexity to capture detailed relationships within the data. In summary, to maximize the effectiveness of neural networks in radiative cooling, proper dataset preparation, architectural optimization, and mitigation strategies for overfitting and underfitting are essential, ensuring reliable predictions and robust material designs.

#### 3.4.2 Deep reinforcement learning

Deep reinforcement learning (DRL) is a powerful approach for optimizing radiative cooling materials, enabling autonomous material selection and structural refinement without the need for predefined design rules or exhaustive simulations. Unlike conventional machine learning models, DRL continuously interacts with the environment, making iterative decisions based on a reward-driven framework to explore and optimize high-dimensional design spaces efficiently.

DRL has been leveraged to optimize emissivity engineering for various radiative cooling applications. One study implemented a deep Q-learning network (DQN) to design wavelength-selective thermal emitters for applications such as thermal camouflage, radiative cooling, and gas sensing [233], as shown in Figure 7B. The model autonomously selected materials and optimized multilayer structures to achieve target emissivity spectra, offering a generalizable framework without prior knowledge of material properties. Another study employed a DQN-based optimization framework to develop a transparent radiative cooling meta-glass, balancing visible transparency, near-infrared reflectance, and mid-infrared emissivity [245]. By optimizing a five-layer dielectric multilayer structure, the model enabled the fabrication of a polymer-free meta-glass with 86 % visible transparency and 89 % atmospheric window emissivity, achieving a temperature reduction of up to 12.7 °C compared to uncoated glass.

DQN and DRL offer a powerful framework for optimizing radiative cooling materials by autonomously exploring high-dimensional design spaces and efficiently identifying optimal structures without relying on predefined rules or exhaustive simulations. Their key advantages include the ability to handle multiobjective optimization, adapt to diverse design problems, and reduce computational overhead compared to brute-force or heuristic methods. Unlike traditional gradient-based approaches, DQN is not constrained by local minima, making it well-suited for complex, nonlinear problems. However, balancing exploration and exploitation remains a challenge, and the black-box nature of reinforcement learning makes interpreting results difficult.

#### 3.4.3 Tandem neural networks and hybrids

Tandem neural networks (TNNs) integrate forward modeling and inverse design into a single framework, where a forward model is trained first to predict optical and thermal properties from structural parameters. This trained model then guides an inverse model to map desired spectral responses to feasible structural parameters. This approach ensures physical readability and fabrication compatibility while significantly reducing computational costs.

One study employed a TNN to design colored daytime radiative coolers (CDRCs) using metal–insulator–metal (MIM) multilayers, where the forward model learned the relationship between MIM structures and spectral reflectance, and the inverse model predicted optimal configurations for targeted emissivity and on-demand color generation [246]. The designed CDRCs exhibited cooling powers ranging from 11.2 to 38.2 W m^−2^ while maintaining vibrant subtractive primary colors, making them suitable for applications in optoelectronics and aesthetics. Another study applied TNN to develop transmissive radiative cooling films with integrated color filtering, where a memetic optimization algorithm first refined the multilayer selective emitter, followed by TNN-based inverse design of nanocavity structures for precise color tuning, as shown in Figure 7C [234]. The forward model accurately predicted spectral responses, while the inverse model optimized structural parameters to balance cooling efficiency and color selectivity, enabling a film that achieved near-ideal emissivity in the atmospheric window alongside high transmissivity for solar energy applications.

While TNNs have proven effective in radiative cooling design, they complement rather than replace classical optimization methods. For instance, one study combined a neural network with a genetic algorithm, where the forward model predicted spectral emissivity, and the genetic algorithm conducted inverse design to maximize emissivity tunability across phase transitions [247]. Expanding on these advancements, another work introduced a hybrid MLP-CNN model incorporating positional encoding to enhance inverse design efficiency [248]. This approach leveraged the MLP to capture global relationships between structural parameters and optical responses, while the CNN extracted spatial features to improve prediction accuracy. The incorporation of positional encoding accelerated convergence and reduced error, significantly improving the design of radiative cooling materials.

## 4 Manufacturing and application

### 4.1 Manufacturing

Numerous studies have been developed to manufacture radiative cooling materials, focusing on enhancing their capacity to reflect sunlight, emit heat efficiently, and support large-scale production. To achieve these goals, various fabrication methods have been proposed to enhance cooling performance while ensuring durability and cost-effectiveness. Depending on the approach, different materials and structural designs are used to maximize thermal management capabilities. In this section, we categorize fabrication methods into four main subsections, with a specific manufacturing approach: multilayer structures, radiative cooling paints, and polymer-based structures.

#### 4.1.1 Multilayer and patterned structure

**Figure 8:**
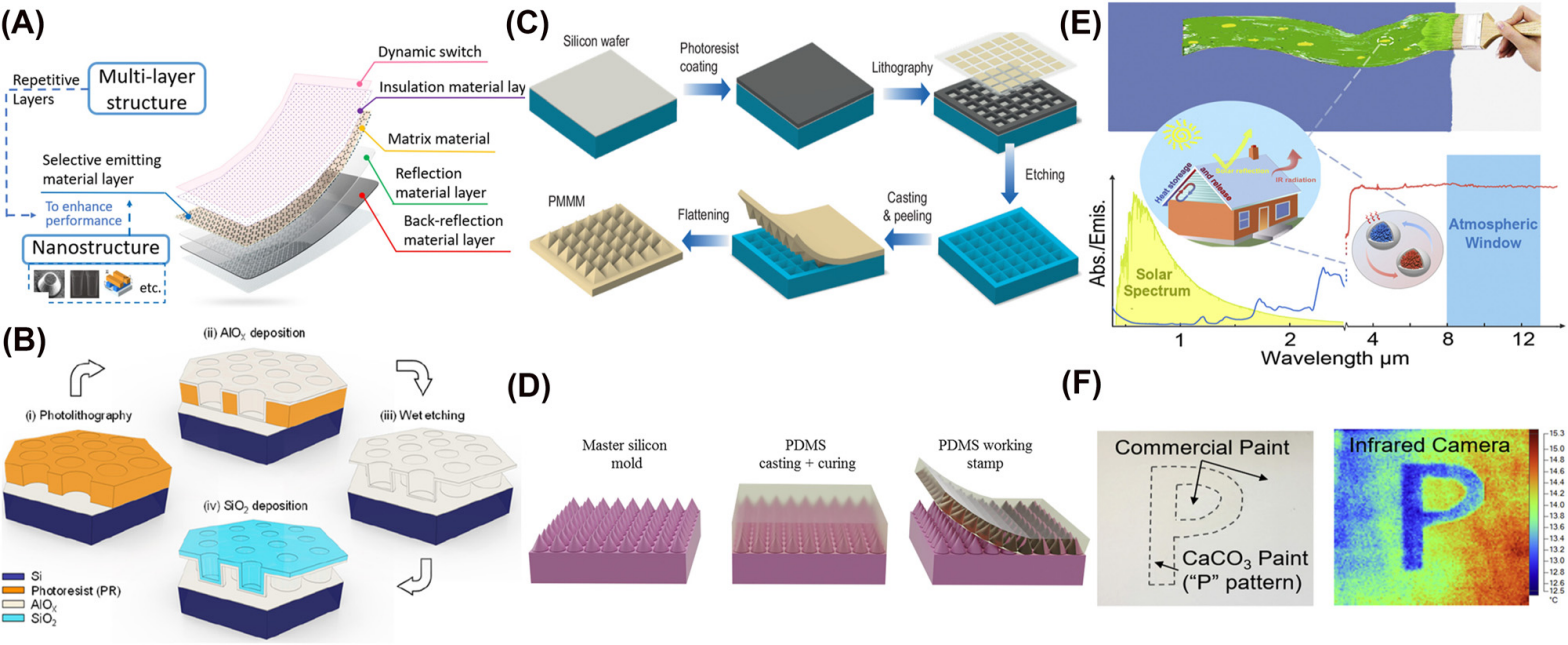
Manufacturing for radiative cooling: multilayer structure (A–D) and paint (E–F). (A) Schematic of a multilayer structure for selective emission and reflection management [249]. Reprinted with permission, Copyright 2020 Elsevier. (B) Schematics of fabrication process of hexagonally arrayed SiO_2_/AlO_*x*_ double-shell hollow cavities [83]. Reprinted with permission, Copyright 2020 American Chemical Society. (C) Schematic of fabrication process of photonic multifunction metamaterials [250]. Licensed under CC BY 4.0. (D) Schematic of the PDMS stamp fabrication for micropatterning heat pipes [251]. Reprinted with permission, Copyright 2023 Elsevier. (E) Bifunctional paint with phase-change microcapsules for enhanced passive cooling [92]. Reprinted with permission, Copyright 2022 Elsevier. (F) CaCO_3_–acrylic radiative cooling paint: spectral emissivity and thermal imaging contrast compared with commercial white paint [94]. Reprinted with permission, Copyright 2020 Elsevier.

A multilayer structure consists of stacked thin layers of materials with different optical properties, optimizing radiative cooling by enhancing thermal emission in the MIR while minimizing solar absorption through strong reflectivity. As radiative cooling technology advances, multilayer structures are being developed with more sophisticated designs to further improve cooling performance, as illustrated in Figure 8A [249]. Due to these advancements, fabrication techniques are evolving to precisely control layer properties, enhancing cooling performance and applicability.

Multilayer fabrication can be broadly categorized into thin-film deposition methods and structural patterning techniques. Thin-film deposition methods, including Physical Vapor Deposition (PVD) and Chemical Vapor Deposition (CVD), are primarily used to form uniform material layers on a substrate. PVD relies on physical processes such as sputtering [84], where high-energy ions eject atoms from a target, and evaporation, where thermal or electron beam heating vaporizes material for deposition [84], [252], [253]. Simple evaporation-based metallization techniques have been widely used for radiative cooling and can achieve optical performance comparable to that of more complex photonic structures [19], [28], [75], [254], [255].

On the other hand, CVD utilizes chemical reactions of gaseous precursors to deposit thin films, enabling high-quality coatings [256], [257], [258], [259], [260]. Several CVD methods, such as Low-Pressure CVD (LPCVD) [261], [262], [263], Plasma-Enhanced CVD (PECVD) [264], [265], and Metal–Organic CVD (MOCVD) [266], [267], [268], [269].

Multilayer fabrication generally encompasses both the deposition of thin films and the patterning required to achieve the desired structural and functional properties. While thin-film deposition methods focus on forming uniform material layers, additional techniques can be applied to create specific patterns and structures. To achieve this, various patterning methods, Electron Beam Lithography (EBL), Photolithography, and Nano-Imprint Lithography (NIL), can be applied. EBL is a high-resolution fabrication method that forms nanostructures by directly patterning a substrate with a focused electron beam [270], [271], [272]. For example, metal–dielectric hybrid structures have been fabricated using EBL and electron-beam evaporation [85]. In this process, EBL was used to pattern cylindrical holes in a PMMA resist on a 150 nm Al-coated silicon substrate, which were then filled with 14 alternating layers of germanium and aluminum through electron-beam evaporation, forming conical metal–dielectric metamaterial structures. Additionally, the radiative cooling metasurface has been fabricated using EBL to define the pattern on a phosphorous-doped n-type silicon wafer. After patterning, plasma-enhanced deep reactive ion etching (DRIE) was employed to carve out the dielectric resonator (DR) structures while preserving their connectivity to the substrate, ensuring structural stability [273].

Photolithography is another fabrication method that enables precise film patterning in the fabrication of multilayer structures [274], [275], [276], [277]. This technique relies on a photomask to project patterns onto the resist using UV light, in contrast to EBL, which performs direct patterning without a mask. For instance, in Figure 8B, hexagonally arranged hollow cavities with a SiO_2_/AlO_*x*_ double-shell layer have been fabricated using photolithography to enhance radiative cooling [83]. The process involves photoresist patterning via a mask aligner, followed by wet etching to remove the photoresist while minimizing stress and preventing fractures. Beyond hollow cavities, photolithography has been employed to create various nanoscale architectures. For instance, a multilayer SiO_2_ micrograting structure has been fabricated to enhance infrared emissivity while maintaining high solar transparency [38] using photolithography. The fabrication process includes SiO_2_ film deposition on a heavily doped Si wafer, followed by photoresist coating and photolithography to define the grating pattern. The process involves SiO_2_ deposition on a doped Si wafer, photolithography to define the grating pattern, reactive ion etching (RIE) for selective removal, and final photoresist removal to complete the micrograting system. In Figure 8D, microphotonic multifunction metamaterials were fabricated by creating a silicon mold with inverted micropyramids through photolithography and etching [250].

Nanoimprint Lithography (NIL) is a high-resolution nanopatterning technology that creates nanometer-scale structures by mechanically imprinting a specialized mold (stamp, template) onto a deformable material, such as polymer, sol–gel, or resist, followed by thermal, UV, or chemical curing to fix the pattern in place [278], [279], [280], [281]. For example, NIL has been applied to polymer-based films by imprinting diffractive grating patterns onto flexible and stretchable substrates for colored radiative cooling applications [282]. In another study, NIL fabrication creates nanostructure patterns on a photoresist-coated glass substrate using laser-induced patterning processes. These patterns are then replicated onto a flexible PDMS stamp and transferred to a PET substrate using UV-curable adhesive, forming photonic crystal structures [16]. Additionally, high-quality glass featuring a nanopyramidal coating was fabricated by depositing a SiO_2_-based sol–gel solution onto the glass substrate, followed by UV nanoimprint lithography (UV-NIL) during the drying phase to imprint uniform nanoscale pyramidal structures. Subsequent annealing solidified these structures, resulting in robust nanopyramidal-coated glass with an optimized spectral profile [283]. In Figure 8D, a flexible PDMS mold, prepared by casting onto a silicon master, was used to imprint nanoscale patterns onto resist-coated copper heat pipe surfaces via NIL. The mold was wrapped tightly around the coated surfaces, ensuring uniform pressure and complete resist filling into its nanoscale cavities. Using a custom UV-NIL setup, the resist was uniformly cured by UV exposure. Afterward, the mold was peeled off, leaving a precise nanoscale pattern imprinted onto the copper surface, enhancing its radiative properties [251].

#### 4.1.2 Paint

Radiative cooling paint is a scalable approach to radiative cooling, making it suitable for broad applications, including buildings [49], [284] and automobiles [285]. When making radiative cooling paint, appropriate particle size [58], broad particle size distribution [58], particle concentration [94], and pigments with wide bandgaps [155] need to be considered for improving solar reflectance. To develop radiative cooling paint with optimal performance, the fabrication process needs to be carefully designed, considering the aforementioned factors.

Recent studies have provided detailed fabrication procedures for optimizing material composition and structural integrity to enhance radiative cooling effects. In Figure 8E, a radiative paint has been presented with latent heat storage using phase-change material-based microcapsules with acrylic resin to enhance cooling. Its temperature measurements confirm its superior cooling performance, including greater temperature reduction and extended heat buffering compared to pure radiative cooling paint [92]. Similarly, a 150 µm thick BaSO_4_ particle film on a silicon wafer has been fabricated to enhance outdoor durability. BaSO_4_ was incorporated into an acrylic-based filler-matrix composite. A high filler volume concentration (60 %) of BaSO_4_ was used to enhance scattering. A broad particle size distribution (398 ± 130 nm) was selected to maximize solar reflectance, outperforming uniform distributions. The addition of an acrylic binder improved structural integrity, ensuring greater reliability in practical applications [58].

Additionally, there has been other research on acrylic radiative cooling paint due to its versatility, toughness, suitable optical properties, and cheap cost [93]. For instance, BaSO_4_ nanoparticle–acrylic paint has been fabricated for high solar reflectance and durability. Acrylic resin was used as a binder for dispersion and adhesion, dissolved in dimethylformamide for uniform mixing. BaSO_4_ nanoparticles (60 %) were added and dispersed via sonication to prevent aggregation. For film fabrication, the paint was applied to cotton pulp paper and cellulose acetate sheets using a calibrated block scraper (20 µm wet layers) [59]. Similarly, CaCO_3_–acrylic paint was fabricated for high solar reflectance and infrared emissivity. CaCO_3_, with its high electronic band gap, minimizes UV absorption. To enhance scattering and reflectance, a 60 % particle volume concentration was used, exceeding the critical particle volume concentration, which improves photon scattering through air void formation. A broad particle size distribution was selected to optimize scattering across the solar spectrum, while the acrylic matrix provided structural stability and infrared emissivity for heat dissipation [94]. In Figure 8F, there is a “P” pattern painted with CaCO_3_–acrylic paint on a surface covered with commercial white paint, placed under direct sunlight. While the pattern appears nearly invisible to a regular camera, an IR camera clearly distinguishes it due to the lower temperature of the CaCO_3_–acrylic paint [94].

To implement radiative cooling paints, recent studies have explored the development of polymer-based paints [172], [286]. A simple, cost-effective, and scalable phase inversion-based method was introduced for fabricating hierarchically porous paint with excellent cooling capability. This method induces controlled phase separation by applying a precursor solution of poly (vinylidene fluoride-co-hexafluoropropylene), nonsolvent water, and acetone as a thin film on a substrate. As acetone rapidly evaporates, micro- and nanopores form, creating an interconnected porous network that enhances thermal emittance and optical performance by efficiently backscattering sunlight [60].

The fabrication process for the fumed silica-added P(VdF-HFP) porous polymer coating has been developed using a combination of solution preparation, phase separation, and controlled drying for improved scalability and structural optimization [287]. In this method, P(VdF-HFP) and fumed silica are first dispersed in acetone to ensure uniform mixing, followed by the gradual addition of deionized water to induce phase separation and form a porous structure. [287]. The MgO–PVDF nanocomposite has been fabricated using a solution-based method to enhance radiative cooling performance. PVDF was dissolved in a solvent, ensuring smooth film formation and high infrared emissivity, while MgO nanoparticles were dispersed via stirring and sonication to enhance solar reflectance by preventing agglomeration. The solution was drop-cast onto a silicon wafer for precise thickness control, followed by thermal treatment to ensure film stability and long-term cooling efficiency [286].

**Figure 9:**
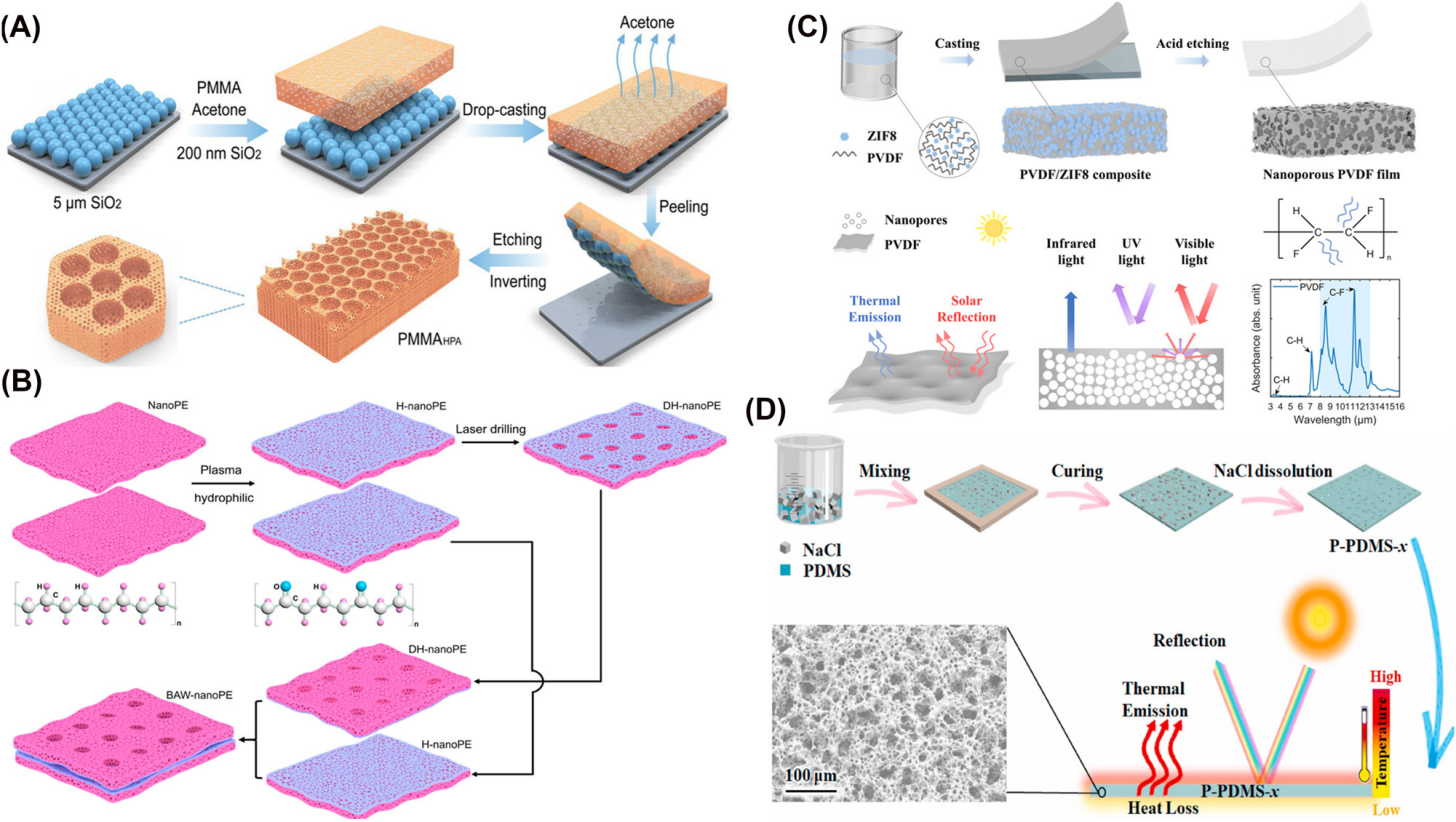
Manufacturing procedure for polymer-based structures. (A) Schematic illustration of the fabrication process of PMMA with a hierarchically porous array [288]. Licensed under CC BY 4.0. (B) Fabrication process and structure of Bilayer Asymmetric Wettability-nanoPE (BAW-nanoPE) [289]. Reprinted with permission, Copyright 2022 American Chemical Society. (C) Schematic representation of the fabrication process and radiative heat transfer mechanism of the nanoporous PVDF_NP_ film [43]. Reprinted with permission, Copyright 2024 American Chemical Society. (D) Schematic illustration of the fabrication process and radiative cooling mechanism of P-PDMS-x films [44]. Reprinted with permission, Copyright 2021 Elsevier.

#### 4.1.3 Polymer-based structure

Polymer-based structures have gained attention due to their lightweight [290], [291], cost efficiency [292], [293], [294], and scalability [295], [296], [297], [298]. This structure has been identified as promising radiative cooling materials to offer a sustainable and energy-saving solution with broad infrared emission and negligible solar absorption [282], [299], [300], [301], [302], [303], [304]. Among the various polymer-based materials studied, this section particularly reviews polymethyl methacrylate (PMMA), polyethylene (PE), polyvinylidene fluoride (PVDF), polydimethylsiloxane (PDMS), and polyethylene terephthalate (PET).

First, the radiative cooling film made of PMMA has been fabricated using the blade-coating method to maximize solar reflection and infrared emission. The key materials included SiO_2_ microspheres, rutile TiO_2_, PMMA, and silane-69. SiO_2_ forms a porous structure that enhances infrared emission, while rutile TiO_2_, with its high refractive index (2.73), increases solar reflectance and improves cooling performance. During fabrication, SiO_2_ was mixed with silane-69 to improve dispersion, and PMMA was dissolved in a solvent to prepare the binder solution. It is reported that PMMA enhances mechanical strength, prevents cracking, and facilitates infrared emission by forming internal voids [305]. In Figure 9A, a scalable and cost-effective manufacturing process was developed to fabricate porous PMMA films for radiative cooling. The process employs a SiO_2_ microsphere template to form a hexagonally packed monolayer on a PDMS-coated glass substrate via a simple rubbing technique. A PMMA–acetone–SiO_2_ dispersion is then drop-cast onto the template, and after solvent evaporation, the film is peeled off. The SiO_2_ template is subsequently removed by hydrofluoric acid etching, yielding a porous PMMA film with controllable thickness (2–160 µm) and tunable optical properties [288].

Beyond PMMA-based radiative cooling films, polyethylene (PE) has also been explored through diverse fabrication techniques to optimize its properties for advanced thermal management. In Figure 9B, the fabrication process and structure of the Bilayer Asymmetric Wettability-nano polyethylene (BAW-nanoPE) system were developed to achieve directional moisture transport and radiative cooling functionality [289]. In the fabrication, plasma-treated nanoPE membranes become hydrophilic on one side (H-nanoPE). One membrane is laser-drilled to create a hole array, forming Drilled Hydrophilic nanoPE (DH-nanoPE). The hydrophilic sides of H-nanoPE and DH-nanoPE are then attached to create BAW-nanoPE. Similarly, polyethylene fabric was fabricated through melt extrusion to form continuous fibers and multifilament yarns in a sustainable manner. Woven on an industrial loom from linear low-density polyethylene, the textile lacked mechanical reinforcement or chemical treatment. Scanning electron microscopy and microcomputed tomography revealed a densely packed structure with high porosity. The fiber size and plain-woven design optimized moisture transport, fast drying, and passive cooling for enhanced comfort [42].

Expanding beyond PE structure, PVDF-based films have also been investigated for radiative cooling applications. A porous PVDF film incorporating zeolitic imidazolate framework (ZIF-8) nanoparticles was developed to enhance radiative cooling performance, as shown in Figure 9C [43]. ZIF-8, a type of Metal–Organic Framework (MOF), was synthesized by mixing a zinc-based precursor with an organic linker in methanol. To fabricate the porous PVDF film, PVDF powder was mixed with ZIF-8 nanoparticles in a solvent and stirred to ensure uniform dispersion. The mixture was then cast onto a glass substrate, where the solvent was evaporated, forming a solid PVDF/ZIF-8 composite film. Finally, the ZIF-8 nanoparticles were removed using hydrochloric acid, leaving behind a porous PVDF structure. This method provides a scalable approach to producing lightweight, flexible, and thermally efficient polymer films suitable for radiative cooling applications. Similarly, a hierarchical TiO_2_–PVDF fiber film was fabricated using electrospinning for radiative cooling applications. PVDF, selected for its strong infrared emission, was dissolved in a solvent mixture with polyethylene glycol as a dispersing agent to ensure a uniform distribution of titanium dioxide nanoparticles. These nanoparticles, known for their high reflectivity, were added to the solution and stirred to form a homogeneous precursor [306].

Beyond PVDF, polydimethylsiloxane (PDMS) has also been utilized in radiative cooling materials. In Figure 11D, the films are fabricated by mixing PDMS prepolymer with a curing agent and NaCl as a sacrificial template, with the NaCl-to-PDMS ratio adjusted to control porosity. The mixture is then cast into a mold to set the film’s dimensions, followed by curing and NaCl removal through water immersion [44]. In another PDMS manufacturing process, Jiang et al. developed a PTFE/PDMS coating for radiative cooling using a brush-coating method. First, polydimethylsiloxane (PDMS) was dissolved in hexane, and polytetrafluoroethylene (PTFE) powder was gradually added under mechanical stirring to form a uniform suspension. To enhance the coating’s properties, tetraethyl orthosilicate (TEOS) and dibutyltin dilaurate (DBTDL) were introduced into the mixture, which was further stirred for better dispersion. The suspension was brush-coated onto a substrate, forming a self-cleaning PTFE/PDMS coating with radiative cooling properties after curing at room temperature.

Lastly, PET has also emerged as a promising material and has been fabricated for radiative cooling. A simple and scalable fabrication process was established for a colored radiative cooler based on a PET film planar structure. The design consists of a radiative PET layer on top and a metal–insulator–metal (MIM) structure as the color layer at the bottom, optimizing both thermal emission and optical properties for radiative cooling. First, the PET film was ultrasonically cleaned with ethanol and deionized water, then dried with nitrogen gas. Next, a MIM structure – comprising a 20 nm silver layer, an adjustable SiO_2_ layer, and a 100 nm silver layer – was deposited using electron beam evaporation. Finally, the MIM structure was secured with UV adhesive, covered with PET, and cured under UV light to complete the assembly [307]. Aerogels made from PET fibers recycled from plastic bottles have been demonstrated to function as both radiative coolers and thermal insulators. When an object is placed beneath the recycled PET aerogel, it is thermally insulated from the surrounding environment while being cooled below ambient temperature via radiative cooling [308]. A scalable and sustainable fabrication method for porous PDMS sponges, utilizing a sugar sacrificial template for radiative cooling, was first proposed. The sugar, which can be recycled via crystallization, makes the process environmentally friendly and free of toxic chemicals. This solution-based PDMS–sugar mixture is compatible with roll-to-roll coating, allowing for mass production comparable to commercial roofing materials [309].

### 4.2 Application

This section discusses its key applications for radiative cooling, particularly in semiconductor devices, personal comfort, agriculture, and building thermal management. In semiconductor devices, radiative cooling helps manage heat dissipation in photovoltaics (PV) [55], [89], concentrated photovoltaics (CPV) [21], [56], thermophotovoltaics (TPV) [90], [310], and data centers [91]. For personal comfort, radiative cooling textiles and wearable materials provide passive cooling by reflecting solar radiation and enhancing infrared emission, offering temperature regulation for clothing and accessories [10], [179], [311], [312], [313], [314], [315], [316], [317]. In agriculture, radiative cooling techniques, such as cooling mulch and films, reduce heat stress in crops, lower soil temperature, and improve water retention, leading to better plant growth and higher yields [95], [96], [97], [318], [319], [320], [321]. Finally, in building thermal management, radiative cooling materials and coatings enhance energy efficiency by reducing indoor temperatures and cooling loads, contributing to sustainable climate control solutions [2], [49], [88], [322], [323].

#### 4.2.1 Building thermal management

**Figure 10:**
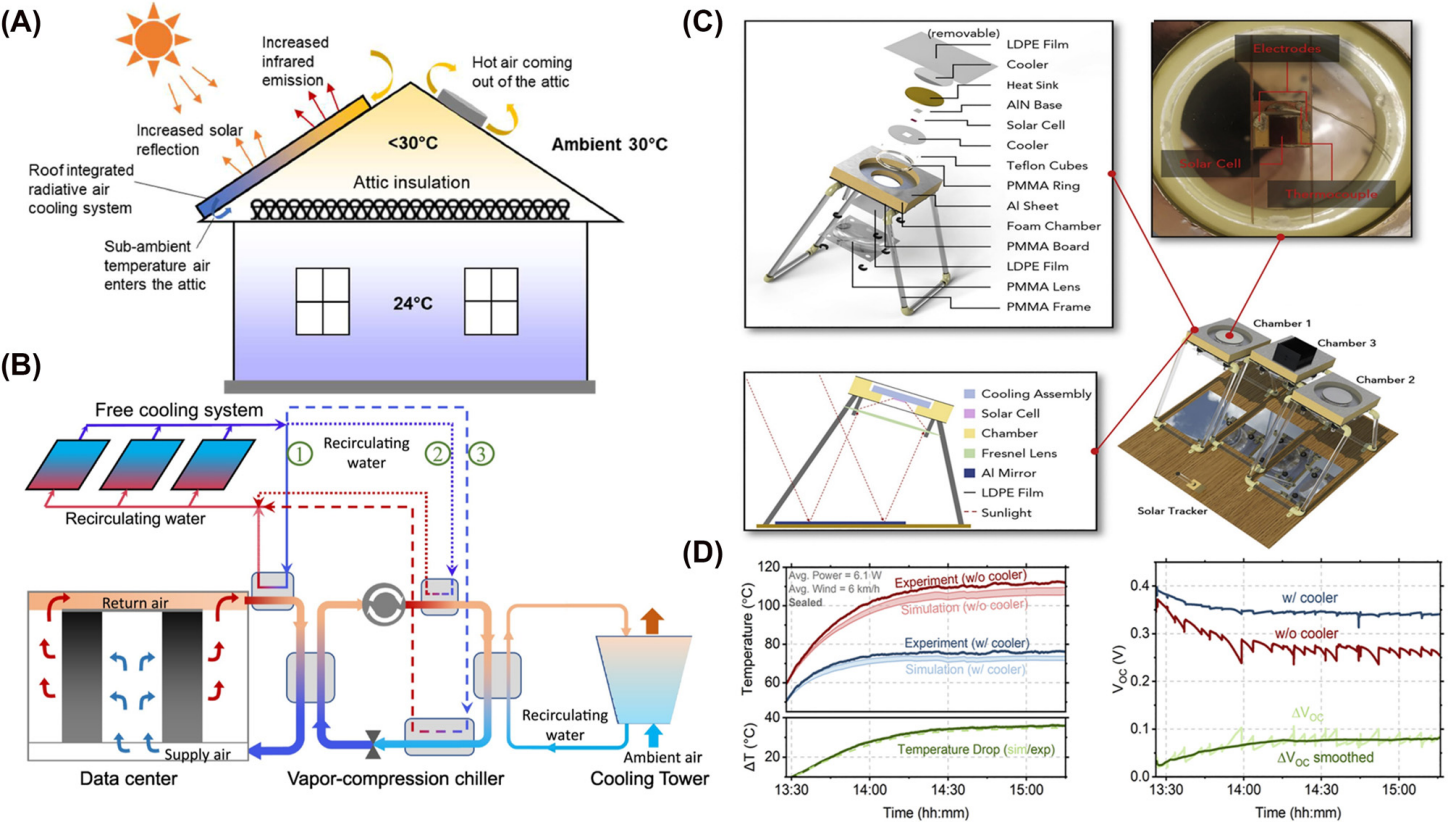
Radiative cooling applications for building thermal management (A), data center (B) and photovoltaic system (C–D). (A) Schematic of a roof-integrated radiative air-cooling system that reduces attic temperature by enhancing solar reflection and infrared emission, keeping the roof cooler than ambient temperature and promoting hot air ventilation [48]. Reprinted with permission, Copyright 2019 Elsevier. (B) Schematic of data center integrated with vapor-compression chiller and cooling tower [91]. Reprinted with permission, Copyright 2024 Elsevier. (C) Schematic of CPV setup with three chamber [21]. Reprinted with permission, Copyright 2020 Elsevier. (D) Temperature and open-circuit voltage profiles with and without a radiative cooler from [21]. Reprinted with permission, Copyright 2020 Elsevier.

One of the most promising uses of radiative cooling is its incorporation into energy-efficient building systems, as it improves energy management and lowers cooling energy consumption. To achieve effective radiative cooling, numerous studies have investigated and proposed a wide range of methods based on air-based systems [48], [324], water-based [325], [326], paints [49], [170], [284], and photonic structures [3], [327], [328], [329], [330]. Figure 10A illustrates a system that integrates radiative sky cooling with attic ventilation. They demonstrate that daytime subambient air cooling of up to 5 °C can be achieved under direct sunlight with airflow. On a typical summer day, attic temperatures are reduced by 15.5–21 °C compared to a shingle roof. Additionally, substantial annual cooling energy savings can be achieved with different attic insulation in various locations [48]. Similarly, an innovative enhancement to conventional building-coating materials has been achieved by integrating particle scattering, sunlight-excited fluorescence, and mid-infrared broadband radiation. This multifunctional approach not only improves radiative cooling efficiency but also enables passive cooling even under direct sunlight. Their upgraded coating, when applied to an aluminum plate, achieved a temperature reduction of 6 °C (7 °C on a scale-model building) below ambient temperature under a solar intensity of 744 Wm^−2^ (850 Wm^−2^), demonstrating a cooling power of 84.2 Wm^−2^ [2]. Additionally, a building-integrated solar heating radiative cooling collector offering both solar heating and radiative cooling was designed to lower installation costs and improve seasonal energy performance. The simulations show 42 % thermal efficiency at zero-reduced temperature and net cooling power over 50 W/m^2^. For a 100 m^2^ building with a 9.43 m^2^ collector, energy savings reach 1.5 kWh more per day than conventional systems. Estimated annual heating and cooling savings are 32.7 % in Madrid, 25.5 % in Tokyo, and 14.0 % in Isfahan [88]. Interestingly, a self-cleaning radiative cooling coating has been developed to enhance building energy efficiency. Their study introduces a modified calcined kaolin/(FEVE-PDMS) coating for building exteriors, designed to effectively reflect and emit heat for temperature reduction. With a high solar reflectance of 92.5 % and MIR emissivity of 94.6 %, the coating efficiently regulates heat while exhibiting superhydrophobic properties, preventing water absorption and minimizing contamination and corrosion. It also demonstrates strong durability against UV aging and mechanical damage. Under a solar irradiance of 742.78 W/m^2^, the coating achieved a temperature reduction of up to 13.12 °C when paired with a PE film and 3.09 °C without shielding [49]. A novel approach known as the equivalent-sky-radiative temperature was proposed to enable more precise simulation of the thermal performance of radiative cooling materials. Integrating this new parameter into building energy simulation tools is expected to enhance computational accuracy and reduce processing costs. Case study results indicate an average error of 5.47 W/m^2^, and the equivalent-sky-radiative temperature data generated for 50 cities worldwide demonstrate its applicability in assessing the thermal performance and energy-saving potential in real buildings [322].

While rooftop-based radiative cooling has advanced significantly, recent efforts have extended its application to vertical walls. For instance, micro-patterned directional emitters with ultrabroadband, angle- and azimuth-selective emissivity have been introduced for passive seasonal thermoregulation. Outdoor tests show temperature reductions of 1.53–3.26 °C in hot weather and increases up to 0.46 °C in cold weather, with cooling and heating powers reaching 40 and 30 W/m^2^, respectively [331]. Similarly, subambient daytime radiative cooling on vertical surfaces has been demonstrated using a hierarchically structured, angularly asymmetric, spectrally selective emitter. Under 920 W/m^2^ sunlight, it cooled 2.5 °C below ambient, outperforming a silica–polymer cooler and white paint by 4.3 °C and 8.9 °C, respectively [332]. A zigzag-based design with asymmetric emissivity enabled radiative cooling for vertical walls, achieving a daily average temperature reduction of 2.3 °C, and up to 3.1 °C under 56 °C ground conditions, corresponding to 67 W/m^2^ of cooling power [323]. A micro-wedge-based directional thermal emitter with angle-selective emissivity has been developed to address temperature inhomogeneity on building facades exposed to both sky and ground. By controlling the direction of thermal emission, it enhances radiative cooling toward the sky while suppressing radiation toward the ground, achieving up to 2 °C lower daytime temperatures compared to isotropic emitters. As a result, it enables 10–40 % cooling energy savings and improves the efficiency of indoor radiant heating [333].

#### 4.2.2 Semiconductor device

As semiconductor devices continue to advance, efficient management of heat dissipation has become a critical challenge for maintaining performance and longevity. This approach is especially beneficial for applications with high thermal loads and strict energy efficiency demands. The appealing candidate is a device with intensive heating and a significant need for energy efficiency. In this section, we introduce photovoltaics, concentrated photovoltaics, thermophotovoltaics, and data centers. Photovoltaics stand out as a relevant example, as their performance and longevity are highly sensitive to temperature variations. The temperature effect can have a negative impact on photovoltaics, leading to accelerated degradation resulting in a decline of power output from the initial rated capacity [334], [335], [336], a decrease in cell efficiency due to reduced open-circuit voltage [337], and a shortened lifespan of the photovoltaic cell [338]. To address these challenges, effective temperature management in photovoltaics is significant for enhancing their performance and lifespan. In this regard, radiative cooling can be beneficial by dissipating excess heat, thereby improving its efficiency. Numerous researchers have explored various structural modifications and material designs to optimize thermal regulation while enhancing light absorption for energy conversion in photovoltaic applications.

For instance, a photovoltaic radiative cooling system was developed to simultaneously generate electricity via photovoltaic conversion during the day and harvest cooling energy through radiative cooling. They designed a 1D multilayer stack and a 2D photonic crystal structure to maximize solar transmittance in the 0.3–1.1 µm range, selectively reflect unnecessary solar radiation, and enhance radiative cooling. When applied to a monocrystalline silicon solar cell, this structure achieved a daytime electricity output of 99.2 W m^−2^ and a nocturnal radiative cooling power of 128.5 W m^−2^, leading to 6.9 % and 30.5 % performance improvements over a bare cell, respectively [89]. To improve the cooling of vertical PV modules, a V-shaped structure was developed to utilize thermal radiation from both sides. As a result, a temperature reduction of 10.6 °C was observed under 1 sun illumination in a controlled indoor environment. Furthermore, field tests conducted in the warm and humid conditions of Thuwal in Saudi Arabia demonstrated that the operating temperature was 0.2 °C lower, efficiency increased by 15 %, and power output improved by 16.8 % compared to conventional horizontal PV modules [55].

**Figure 11:**
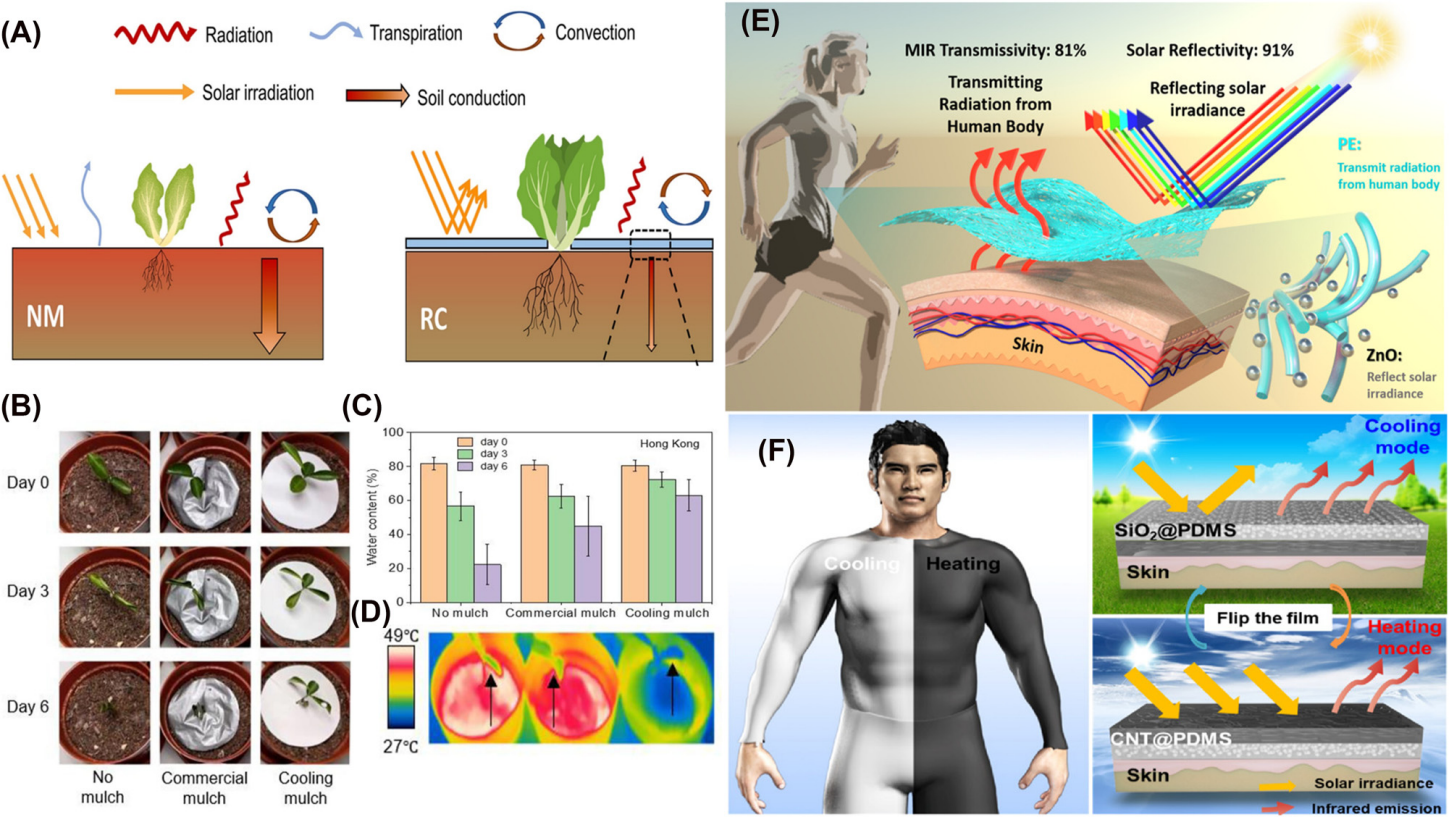
Radiative cooling applications for agriculture (A–D) and personal comfort (E–F). (A) Schematic of soil condition under sunlight: comparison of soil condition without (left) and with radiative cooling coverage (right) and its effect on mulch. No Mulch, denoted as “NM,” stands for soil left exposed without surface covering, while “RC” refers to radiative cooling [95]. Licensed under CC BY 4.0. (B) Pictures of growing seedlings on different days across three experimental setups in Hong Kong [318]. Reprinted with permission, Copyright 2024, Elsevier. (C) Shows the water content of each mulching group, while infrared images of these groups are presented in (D) [318]. Reprinted with permission, Copyright 2024, Elsevier. (E) Nanoparticle-doped wearable textile for enhanced personal comfort through high solar reflectivity and MIR transmissivity. Reprinted with permission from [10]. Copyright 2022 American Chemical Society. (F) A dual-mode film for adaptive personal thermal management. The SiO_2_ side enables radiative cooling by reflecting solar radiation, while the CNT side enhances solar absorption for heating [342]. Reprinted with permission, Copyright 2022 American Chemical Society.

In addition to these studies, further efforts have been made to enhance photovoltaic performance by integrating radiative cooling into concentrated photovoltaic (CPV) systems. For example, radiative cooling simulations were performed under varying heat load conditions (6 W–100 W), incorporating different CPV cooling designs. In Figure 11C, Chamber 1 contains a solar cell with two soda-lime glass radiative coolers, while Chamber 2 has a similar structure but replaces the coolers with aluminum reflectors to minimize solar heating. Both chambers include removable LDPE films to simulate different convection conditions. Chamber 3 is equipped with a thermal power sensor to monitor incident solar power [21]. In Figure 11D, the left figure shows the measured and simulated temperatures of solar cells in Chamber 1 (blue) and Chamber 3 (red), with the bottom graph showing the temperature drop (green) due to cooling. Their cooling design led to temperature reductions ranging from 5 °C to a maximum of 36 °C, along with an 8 %–27 % increase in the open-circuit voltage of GaSb solar cells. Additionally, high-concentration photovoltaic (HCPV) systems incorporating radiative cooling were investigated to evaluate their thermal and electrical performance. A proposed cooler design, composed of low-iron soda-lime glass with a porous antireflection layer and a diamond heat spreader, enables strong MIR emittance and high solar transmission, allowing effective cooling under direct sunlight with minimal losses in concentrated solar irradiance. Simulations show that the proposed design lowers temperature by 14 K, requires less surface area for steady-state operation at 333.15 K, and achieves a cooling power per weight of 75 W/kg, 3.7 times higher than a conventional copper cooler [56].

In addition to CPV applications, radiative cooling has also been explored in thermophotovoltaic (TPV) systems, which convert thermal radiation into electricity. The use of radiative cooling to mitigate heating effects on the PV diode in a TPV system has been investigated. By optimizing the area ratio between the cooling emitter and the PV diode, below-ambient cooling has been achieved, leading to improved TPV conversion efficiency. Their simulations with realistic materials suggest that low-iron soda-lime glass, further enhanced with photonic crystal (PhC) structures, can significantly lower the PV diode temperature, resulting in up to an 18 % efficiency increase and a 2,200 % improvement in mean time between failures [90]. Similarly, a TPV system integrating a metamaterial thermal emitter (MTE) and a broadband passive radiative cooler (PRC) has been demonstrated. The MTE emits within the optimal wavelength range of 0.8–1.72 µmm, matching the external quantum efficiency of GaSb PV cells and achieving 36.55 % efficiency at 1400 K without cooling. When integrated with the PRC, the system’s operating temperature is reduced by 44 K, resulting in a 1.89 % increase in efficiency [310].

Several studies have explored innovative implementations of radiative cooling beyond photovoltaics. For example, there is a pioneering study that has successfully utilized radiative cooling strategies, particularly in data centers. In Figure 11, three different integration strategies were obtained. In Case 1, cooling water from the radiative free cooling system precools warm return air from the data center using a water-to-air heat exchanger. In Case 2, the cooling water precools compressed refrigerant before condensation through a water-to-gas heat exchanger. Lastly, the cooling water further cools condensed refrigerant before expansion via a water-to-liquid heat exchanger in Case 3. They found different degrees of energy and water savings depending on the implementation method. In particular, direct cooling of return air demonstrated a 20.0 % annual energy reduction in tropical climates. Meanwhile, radiative cooling of refrigerant before reaching the chiller resulted in an 84.0 % reduction in water consumption on average [91]. The concept of “concentrated radiative cooling” has been demonstrated as a viable approach in recent work. In this approach, a radiative cooling system is placed within a MIR reflective trough, allowing the lower surface – previously unused for radiative cooling – to emit heat to deep space via the reflective trough. Their field experiments show that the trough-enhanced radiative cooling pipe achieves over twice the temperature reduction of a standalone pipe. As a precooling unit in air conditioning, it could cut electricity use by 75 % in Phoenix, Arizona, and 80 % in Reno, Nevada [339].

There have also been other innovative studies demonstrating the use of radiative cooling to power an LED to present its potential as a low-cost, off-grid solution for generating electricity – and light – from the thermal contrast between Earth and outer space. A thermoelectric generator was designed with its cold side facing the night sky, leveraging radiative sky cooling to lower its temperature several degrees below ambient. The warm side, exposed to the surrounding air, remained at ambient temperature, creating a temperature difference across the device. This difference enabled night-time power generation of approximately 25 mW m^−2^, which is sufficient to illuminate a low-power LED. The study further identified feasible pathways to enhance performance to over 0.5 W m^−2^ using commercially available components [340]. Similarly, an outdoor LED streetlight demonstrates how radiative cooling can be effectively applied for thermal management. Using a sky-facing design with nanoPE – a material that is transparent to infrared and reflective to visible light – the system passively dissipates heat to the sky. This approach reduced LED temperatures by 7.8 °C in lab settings and 4.4 °C outdoors, resulting in efficiency improvements of 5 % and 4 %, respectively. When applied on a national scale, this translates to estimated 1.9 TW-hours of annual energy savings and a reduction of approximately 1.3 million metric tons of CO_2_ emissions in the United States [341].

#### 4.2.3 Agriculture

Heat stress is a major challenge for crop production, especially in regions with high temperatures, as it can severely impact crop yield, quality, and overall plant health. Plants are especially vulnerable to heat stress, which negatively impacts various stages of their growth including photosynthetic efficiency, water balance, nutrient absorption, respiration, and reproductive processes [343], [344], [345]. As shown in Figure 11A, the schematic on the left shows bare soil exposed to sunlight, where solar irradiation is absorbed, resulting in significant heating for the soil. In contrast, the right schematic shows soil covered with radiative cooling coverage, where the mulch remains at a lower temperature under sunlight, effectively minimizing downward heat conduction into the soil. Consequently, heat-stressed plants exhibit reduced yields [95]. Applying a radiative cooling technique can be a promising solution to alleviate stress due to reducing temperatures without relying on external power sources. By passively dissipating heat, radiative cooling can help maintain optimal conditions for crops, reducing stress and enhancing productivity as well as the overall environment for crops. There have been extensive efforts to improve the agricultural environment for sustainability and productivity through radiative cooling. For example, a soil cooling strategy using radiative cooling mulch has been proposed, as briefly shown in Figure 11A [95]. Radiative cooling mulch enables effective soil cooling without additional energy consumption by reflecting a high percentage of sunlight and emitting strong thermal radiation. Experimental results show that applying this technology reduces root zone temperature by 12.5 °C and increases summer crop yields by 127.4 %. Lin et al. [318] proposed the biodegradable ethyl-cellulose (EC) radiative cooling film with substantial advantages in reducing heat stress in plants. In Figure 11B, seedlings grown with cooling mulch exhibit the most growth over time compared to other conditions. The film effectively decreases soil temperature by 50 %, minimizes soil moisture evaporation by 60 % (Figure 11C), and supports leaf transpiration. In Figure 11D, infrared images illustrate that the cooling mulch group, located on the far right, exhibits a significant cooling effect.

In addition to lowering soil temperature, recent studies have also explored the use of radiative cooling for water collection [96], [97], [319] to support sustainable agriculture. For example, a photosynthetically active radiative cooling film was proposed to reduce air temperature, minimize water evaporation, and enhance plant growth in dry-land regions. It is composed of a photonic crystal layer, polydimethylsiloxane, and polyacrylamide hydrogel; the film combines high radiative cooling efficiency (92 % MIR emissivity) with selective sunlight transmission (71–77 % for photosynthetically active wavelengths). Field tests showed water evaporation decreases of 2.1–31.9 %, and biomass yield increases of 20–370 % as well as air temperature reductions of 1.9–4.6 °C [320]. Atmospheric water harvesting using radiative cooling fabrics has been explored through a hierarchically structured cellulose network and a hybrid sorption–dewing mechanism. Water adsorption onto the hydrophilic functional groups of cellulose was driven by sorption at low relative humidity (RH) and dewing at high RH. In field tests, the cellulose sample recorded a water uptake of 1.29 kg/kg at 80 % RH overnight [321]. Additionally, water harvesting through nighttime radiative cooling has been studied by extending the functionality of solar panels into the nighttime. By identifying the optimal temperature and humidity range, the water harvesting potential has been analyzed. Additional emissivity engineering improves the water generation rate and suitable environmental conditions, allowing for more efficient water harvesting [346].

#### 4.2.4 Personal comfort

Since there are limitations to modifying outdoor conditions due to factors such as ground thermal radiation, wind conditions, and humidity, advanced textile materials in clothing or the development of smart, wearable, and portable thermal regulation devices offer effective solutions for human body temperature management. These technologies work by directly regulating body temperature through fabric structures that efficiently emit infrared radiation while blocking visible light, providing rapid cooling by acting directly on the skin.

Extensive research has reported its effectiveness in personal thermal management, particularly through IR-transparent radiative cooling [7], [313], [347], IR-emissive radiative cooling [60], [348], [349], [350], [351], and solar-reflective cooling [9], [351]. Building on these characteristics, researchers have developed wearable nanofabric technologies for personal body thermal management. For example, visible-opaque fabrics (ITVOF) can enable passive cooling by allowing the body’s thermal radiation to dissipate directly into the outdoor environment. In their fabric, polyethylene fibers were selected and structurally engineered to reduce infrared reflection while preserving visible opacity [312]. Using polymer structure, nanoporous polyethylene (nanoPE) has been developed as a personal thermal management textile that transmits MIR radiation while blocking visible light. Their nanoPE reduced skin temperature by up to 2.7 °C compared to conventional fabric, cotton [313]. In Figure 11E, a nanofabric with excellent optical properties and practical applicability was developed using zinc oxide (ZnO) and polyethylene (PE)-based nanoparticle-doped polymers processed via electrospinning. Additionally, heat transfer model was established to quantitatively analyze the cooling performance of the fabric under various climatic factors, including solar intensity, ambient temperature, atmospheric radiation, wind speed, and parasitic heat loss rate. The results showed that the nanofabric effectively dissipates body heat, providing additional cooling effects in most environments. Even under extreme conditions, it was proven to be an efficient thermal regulation solution by reducing cooling demand compared to cotton fabric [10]. A nanocomposite textile incorporating zinc oxide-embedded polyethylene was developed for radiative outdoor cooling, capable of reducing simulated skin temperature by 5–13 °C under peak sunlight by reflecting over 90 % of solar irradiance and transmitting body heat [314].

The head and face are frequently exposed to sunlight, causing heat discomfort. As a result, cooling fabrics can be incorporated into hat and mask designs for enhanced comfort. For example, a radiative cooling paper (RCP) hat was developed using SiO_2_ fibers and fumed SiO_2_ for effective head thermal management [179]. The RCP exhibits high solar reflectivity (0.97) and atmospheric window emissivity (0.91), reducing hair temperature by 12.9 °C compared to a white cotton hat. Similarly, A bio-skin-inspired PDRC metafabric with 97 % solar reflectance and high atmospheric emissivity was developed, achieving 12.6 °C subambient cooling. When used in a hat, it kept wearers 16.6 °C cooler than those wearing standard hats [315]. For the mask, a polymer-based composite (PCM) face mask with radiative cooling capability was developed to enhance thermal comfort while maintaining protection against particulate matter and viruses [316]. Unlike conventional masks, which cause discomfort due to heat buildup, the PCM mask incorporates a silver nanoparticle coating. Its outdoor tests with simulated skin showed that the PCM mask reduced temperatures by 4 °C compared to a standard mask. Additionally, a hierarchical polyurethane/metal–organic framework (MOF) composite nanofiber membrane has been developed, combining passive radiative cooling with photocatalytic antibacterial functionality [317]. The mask reduced the temperatures by ∼7.2° C under sunlight and ∼5.5° C at night. Additionally, the ZIF-8 nanoparticles provide 96 % bacterial mortality, making it ideal for medical protective textiles, offering a dual benefit of cooling and infection prevention.

Interestingly, several research groups have explored dual-functional textiles that incorporate both cooling capabilities but also heating functionality to expand their usage depending on seasonality. In Figure 11F, a dual-mode film, referred to as a “Janus” film and composed of PDMS, was developed to enable simultaneous heating and cooling functions. In cooling mode, it consists of the aluminum (Al) backing and embedded silicon dioxide (SiO_2_), resulting in a temperature reduction of approximately 2 °C. In contrast, the structure incorporating carbon nanotubes (CNTs) on the heating side results in a temperature increase of around 7 °C [342]. Similarly, a Janus-structured nanofiber film was fabricated using the fiber-spinning asymmetric chemical assembly (FACA) method for smart temperature regulation. By integrating AgNWs and PVDF-HFP with opposite thermal radiation on rGO film, the material enables dual heating and cooling, with the AgNW side staying 11 °C cooler than the PVDF-HFP side under 1 sun irradiation [352].

However, in urban environments, the effectiveness of cooling textiles can be significantly compromised due to the heat island effect, as they absorb thermal radiation from the ground and surrounding buildings. To address this, some studies have accounted for heat gain from heated urban surfaces. For example, a theoretical model comparing selective long wave IR emitters with conventional broadband emitters showed that selective emitters can provide up to 50 W/m^2^ of cooling in summer and 15 W/m^2^ of heating in winter. These predictions were validated by outdoor experiments, where vertically mounted selective emitters maintained temperatures 0.43 °C–0.86 °C cooler in summer and 0 °C–0.21 °C warmer in winter than broadband emitters. These temperature differences translated into relative heat flow gains of 21 W/m^2^ cooling in hot conditions and 10–30 W/m^2^ heating in cold environments [254]. Similarly, spectrally selective hierarchical fabric (SSHF) has been developed to achieve effective personal radiative cooling even under urban heat island conditions. The fabric selectively emits in the atmospheric transmission window while strongly reflecting solar radiation. Through molecular design and a nano–micro hybrid fibrous structure, the SSHF achieved a high solar reflectance of 0.97. In simulated urban environments, the SSHF maintained temperatures up to 2.3 °C lower than a solar-reflecting broadband emitter when mounted vertically [353].

## 5 Discussion and outlook

Despite substantial advancements in radiative cooling technology design and manufacturing, several challenges must be addressed to fully realize their potential in critical applications. First, it is crucial to apply design strategies that approach the theoretical efficiency of photonic radiative cooling, which imposes constraints on cooling power under practical conditions. The most relevant approaches include physics-driven, biomimetic, computational, and artificial intelligence-driven approaches. More research is required to select the best approach for optimizing the design, materials, and performance of the system, while balancing computational efficiency and feasibility.

In addition to design strategies, real-world implementation poses further challenges. For example, when applied to photovoltaic modules, the cooling effect of radiative coatings remains modest. Although they can slightly reduce module temperature, their overall impact is limited when generating maximum power – particularly when compared to the significantly greater influence of ventilation [354].

Material durability also remains a critical concern, as prolonged exposure to environmental factors such as moisture, dust, and mechanical wear can degrade cooling performance over time. Enhancing material longevity and robustness is essential for facilitating widespread adoption. For example, water-based radiative cooling paints remain a challenge because emulsion-based binders often lead to weak adhesion and poor film formation, while strategies to improve solar reflectivity – such as adding porosity or white pigments – can reduce mechanical strength. Moreover, nanoparticle dispersion in aqueous systems is difficult, further limiting both optical and structural performance [355]. Addressing these issues through novel material design and protective coatings will be crucial for long-term reliability.

Scalability and cost-effectiveness present additional barriers to commercialization. The development of manufacturing processes that enable large-scale production while maintaining performance and affordability is essential for integrating radiative cooling into mainstream applications. Furthermore, climate adaptability remains an ongoing challenge, as performance can be significantly affected by high humidity and persistent cloud cover. Tailored solutions that optimize radiative cooling across diverse climatic conditions are necessary to ensure consistent efficiency. Additionally, emerging hybrid approaches that combine radiative cooling with thermoelectric power generation have shown promise in achieving both thermal management and energy harvesting under variable conditions [356].

In summary, the past decade has seen remarkable progress in radiative cooling research, with innovations in material design and fabrication techniques enhancing its viability as a sustainable cooling strategy. Ongoing efforts continue to refine existing technologies, expand application possibilities, and overcome critical limitations. By addressing these challenges, radiative cooling can play a pivotal role in improving energy efficiency and climate resilience, positioning itself as a key component of future cooling solutions.
